# Synthesis of Imidazolium Salts Linked to a t-Butylcalix[4]arene Framework and the Isolation of Interesting By-Products

**DOI:** 10.3390/molecules30193954

**Published:** 2025-10-01

**Authors:** Michael J. Chetcuti, Rahma Aroua, Abdelwaheb Hamdi

**Affiliations:** 1 Organometallic Chemistry Group, LIMA—UMR CNRS 7042, European School of Chemistry, Polymers and Materials (ECPM), Universities of Strasbourg and of Upper Alsace, 25 Rue Becquerel, 67087 Strasbourg, France; rahma.aroua@gmail.com; 2 LR05ES09 Laboratory of Applied Chemistry and Natural Substances Resources and Environment (LACReSNE), Faculty of Sciences of Bizerte, University of Carthage, Bizerte 7021, Tunisia; abdelwaheb.hamdi@istmt.utm.tn

**Keywords:** calix[4]arene, imidazolium salt, hydrogen bonding, nickel N-heterocyclic carbene

## Abstract

A series of functionalized calix[4]arenes were prepared that contain *mono-* and *bis-*(alkoxy)imidazolium groups that are linked to the lower rim of a t-butylcalix[4]arene framework. These molecules have potential as anion-complexation reagents and as precursors to N-heterocyclic carbene complexes that are attached to a calixarene framework. They were prepared by the preliminary reaction α,ω-dibromoalkanes with the parent t-butylcalix[4]arene to give *bis-*ω-bromoalkoxy groups that are connected to the calix[4]arene framework in the 25- and 27-positions. The reaction of the *bis-*substituted calixarenes with TiCl_4_ led to the removal of one bromoalkoxy group to give *mono-*substituted derivatives. Both the *mono*- and *bis*-functionalized calixarenes were reacted with N-substituted imidazoles to give a series of *mono-* or *bis-*imidazolium salts with the imidazolium group tethered to the calix[4]arene via O–(CH_2_)_n_*–* linkages (n = 2, 4, or 6). Unexpected *bis-*calix[4]arene products, in which the calixarenes are linked together via bridging organic groups, were obtained in some of these reactions. One bridge consists of two calixarenes linked together via two –C_2_H_4_– groups while the other had a –O–C_4_H_8_–imidazolium-C_4_H_8_–O– linker tethering the two calix[4]arenes together. Both these species were characterized by single crystal X-ray diffraction studies. The structures both had significant disorder but, nevertheless, the data do establish their structures. That the imidazolium-substituted calix[4]arene cations are precursors to N-heterocyclic carbene complexes of nickel was demonstrated by the reaction of a *mono-*imidazolium-substituted calix[4]arene with nickelocene to give the fully characterized N-heterocyclic carbene nickel complex linked to the calix[4]arene group.

## 1. Introduction

A lot of our recent work has focused on the chemistry and the catalytic activity of N-heterocyclic carbene complexes of nickel, of generic formula [Ni(h^5^-C_5_H_5_)(NHC)(X)] where NHC = a N-heterocyclic carbene and X = Cl, Br or I [1,2,3,4]. A subset of this research has involved the catalytic potential of these species when they are linked to a calixarene framework [5]. The nickel complexes are prepared in a well-known reaction by treating nickelocene with imidazolium salts [6]. The research reported here describes the synthesis of such salts prior to their conversion to organometallic compounds and the isolation of interesting side products during such syntheses.

Imidazolium groups linked to calix[4]arenes are known, though these groups are usually attached to the upper rim of the calixarene after elimination of the t-Bu groups. Some recent examples include the preparation and use in CuAAC catalysis of carboxytriazolyl amphiphilic derivatives of calix[4]arenes that are prepared via upper rim imidazolium-substituted calix[4]arenes [7], their use in aqueous solution for Suzuki coupling [8], and the synthesis of NHC polymeric particles linked to calix[4]arenes as supports for active palladium nanoparticle catalysts by the same Russian group [9]. Upper rim-substituted calixarenes with imidazolium salts have been used to prepare metal NHC complexes, as the imidazolium salts are precursors to NHC complexes, and these species have found use as catalysts [10,11]. Indeed, the nearby calixarene cavity has been likened to a nanoreactor [12] for these catalysts.

*Bis-* and *tetrakis-*imidazolium salts that are linked to the lower rim of a calix[4]arene have been prepared by us [13], and others [14,15], and catalytic studies have appeared on the use of p-tert-butylthiacalix[4]arene-linked Pd-NHC complexes that are derived from imidazolium groups linked to a p-tert-butylthiacalix[4]arene [16], which are related to what we report in this paper. One such catalytic system, formed in situ, consisted of imidazolium salts, combined with Cs_2_CO_3_ as the base and Pd(OAc)_2_ as the palladium source [17]. These species were used as catalysts for the Suzuki–Miyaura coupling reaction [18,19]. Another example includes the use of a compound that contains *bis*-(methylimidazolium)groups linked to t-butylcalix[4]arene that finds use as both a surfactant and a catalyst for Ullmann coupling in aqueous media [20].

It is possible to make some generalizations that compare and contrast the two types of calixarene substitution. In general, upper rim substitution, i.e., on the *para*-position relative to the phenolic OH group, does not lead to major conformational changes in the calixarene [21], while lower rim substitution can lead to more conformational changes that are probably due to different modes of H-bonding that may be possible on substitution. Nevertheless, we have not seen such conformational changes in our research so far, as all our lower rim-substituted calix[4]arenes retain the cone configuration of their parent. In general, it is easier to prepare lower rim derivatives as the OH or the methylene groups are more reactive [22]. As the phenolic OH groups are more chemical reactive, it is also, in principle, easier to prepare substituted derivatives via O–H bond activation.

Imidazolium containing cationic species that are part of a calixarene have been probed as complexing agents for inorganic anions such as H_2_PO_4_− and HSO_4_−, towards which they exhibit some selectivity [23]. Recently *tetrakis*-imidazolium salts have shown antibacterial activity on par with some antibiotics [24].

This manuscript delineates the synthesis and characterization of a series of mainly *mono*-substituted calixarenes with imidazolium ligands and the isolation of small quantities of unexpected products with interesting structures. The conversion of one of the *mono-*substituted species into a nickel-N-heterocyclic carbene (NHC) complex demonstrates that, in principle, all the new imidazolium salts can be converted in good yields to nickel NHC derivatives. The question of whether functionalizing the lower rim of a calix[4]arene through monosubstitution and subsequently transforming these species into organometallic derivatives enhances or diminishes their catalytic performance remains open. Equally interesting is the question of how the spatial relationship between the calixarene scaffold and the organometallic fragment influences activity. By adjusting the length of the methylene linker, this distance can be systematically varied, yet the precise effect on catalytic potential remains unclear. Subsequent syntheses of nickel NHC complexes that are grafted onto calix[4]arene frameworks, that in principle can be prepared from the imidazolium salts described in this manuscript, should shed light on some of these questions.

## 2. Results

### 2.1. Synthesis of 25,27-Bis(alkoxy)imidazolium t-Butylcalix[4]arenes

**Scheme 1:**
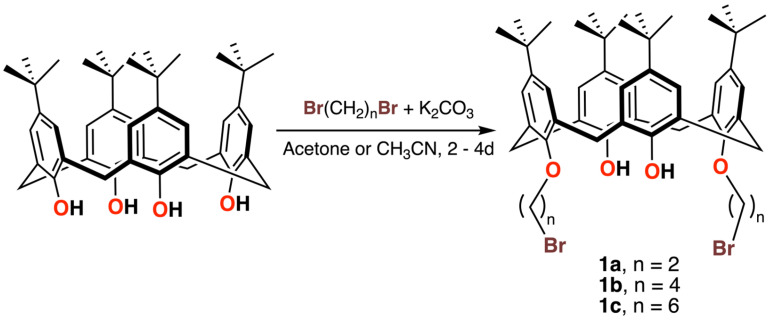
Syntheses of 25, 27-*bis*(ω-bromoalkoxy)t-butylcalix[4]arenes, **1a** and **1b** [13] and **1c**.

t-Buylcalix[4]arene can be functionalized by reaction with α,ω-dibromoalkanes in the presence of a base, as shown in Scheme 1, to give *bis*(alkoxybromo) compounds **1a**–**1c** with varying length methylene spacers that we have previously reported for **1a** and **1b** [13]. The *bis*(hexoxybromo) compound, **1c**, is new and its characterization data are given in the Experimental section. Its ^1^H NMR spectra agree with its proposed geometry, and the data are consistent with a molecular geometry that is similar to what is observed for **1a** and **1b**.

### 2.2. Synthesis and Structure of Compound **2**

**Scheme 2:**
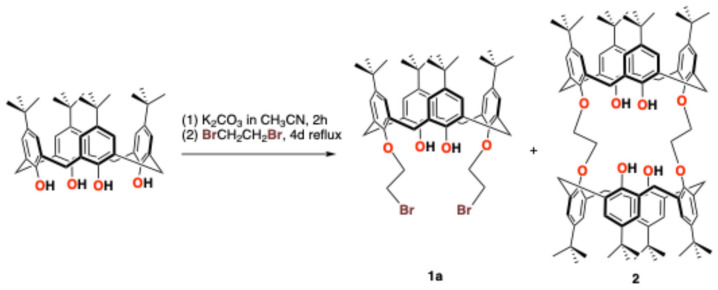
Synthesis of **1a** and compound **2**.

When the synthesis of **1a** was modified (different solvents and conditions were employed compared to its previous synthesis)—see the Experimental section—the yields obtained from **1a** were significantly lower, but a few small crystals (<5 mg, that was not weighed) of another compound (**2**) were isolated as shown in Scheme 2. Under these conditions, a lot of the initial t-butylcalix[4]arene did not react. We were unable to obtain a ^13^C NMR spectrum of this compound, but its ^1^H NMR displays a spectrum of a symmetric molecule. All –OCH_2_CH_2_O– protons are equivalent and appear as a singlet. A mass spectrum of the compound was unfortunately not obtained as the ^1^H NMR tube broke, and the sample was lost.

The bridging methylene groups of the rim of the calixarene appear as a characteristic AB multiplet with a coupling constant of 13 Hz, indicative of a cone conformation for the calix[4]arene. Two different ^t^Bu peaks are observed and the two different kinds of phenoxy protons display two singlets in the ^1^H NMR of **2**. Its structure is shown in Scheme 2.

**Figure 1:**
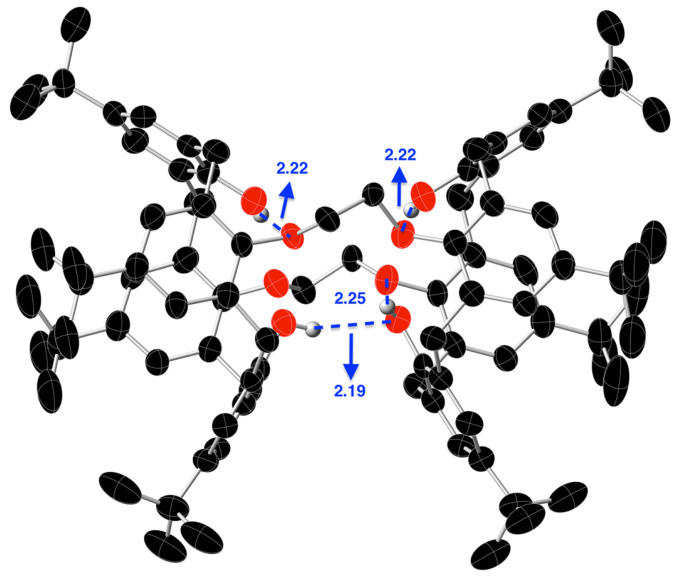
Molecular structure of **2**, with ellipsoids shown at the 50% level. **Carbon** and **oxygen** atoms are color-coded as shown. Only the four **O–H** hydrogen atoms involved in H-bonding are shown, for clarity, as arbitrarily sized gray spheres. The H-bonds are depicted as dotted blue lines; distances are in Å. Just one of the disordered two positions (the major one, when the occupancy is not 50:50) of the t-Bu methyl groups, and of the disordered –C_2_H_4_– group, is shown.

The structure of **2** was established by a single crystal X-ray diffraction study and is shown in Figure 1. There is significant disorder in the unit cell, as is frequently the case with calixarenes, which are relatively large and conformationally non-rigid molecules. Two different crystals were subjected to the X-ray diffraction study, and both gave relatively poor data due to twinning problems and did not give high θ reflections. Compound **2** crystallizes with four molecules of dichloromethane (two of which also exhibit disorder which could not be properly modeled) in the asymmetric unit. Furthermore, many of the t-butyl groups are disordered over two positions. One of the two bridging O–C_2_H_4_–O units is also disordered in a zig-zag arrangement, leaving the oxygen atoms in both disordered chains in the same position. Despite the unsatisfactory data, we believe the structure still establishes the molecule’s structure shown in Figure 1.

There are a number of H-bonded interactions in this molecule. One calixarene exhibits 2 O–H···O bonds of 2.220 and 2.247 Å between the O–H hydrogen atoms and the oxygen atoms of the two ethoxy groups. The other calixarene shows the same kind of hydrogen bond (O–H···O = 2.200 Å) to one of the ethoxy groups, but the other O–H hydrogen interacts with an O–H oxygen atom in the other calixarene (O–H···O–H) = 2.188. These interactions may persist in solution as the OH protons resonate at d = 7.23 ppm in the ^1^H NMR spectrum, which is ≈2–3 ppm upfield compared to their usual positions in di-substituted calix[4]arenes that we have reported earlier [13]. The hydrogen atoms of one of the disordered CH_2_Cl_2_ molecules also probably exhibits a H-bonding interaction with one of the hydroxy group oxygen atoms. These interactions are highlighted in Figure 1.

### 2.3. Synthesis of Mono-Substituted Calix[4]arenes

Mono-substituted (ω-bromoalkoxy)t-butylcalix[4]arene compounds were then targeted. A variety of literature methods exist for the preparation of such species, but some methods we tried did not give good results in our hands. A direct mono-alkylation method consists of reacting the calix[4]arene with an alkylating agent in the presence of a weak base such as CsF [25], Na_2_CO_3_ [26], and K_2_CO_3_ (in solvents such as DMF or acetonitrile). Stronger alkalis such as Ba(OH)_2_ in DMF, or NaH suspended in toluene [27], and NaOMe in CH_3_CN [28] have been proposed. However, attempts to synthesize mono-substituted calix[4]arenes by using the base route led to mixtures of mono- and di-substituted calixarenes as well as unreacted starting material and were unproductive in our hands. Mixtures were obtained that were difficult to separate into their constituents via column chromatography.

**Scheme 3:**
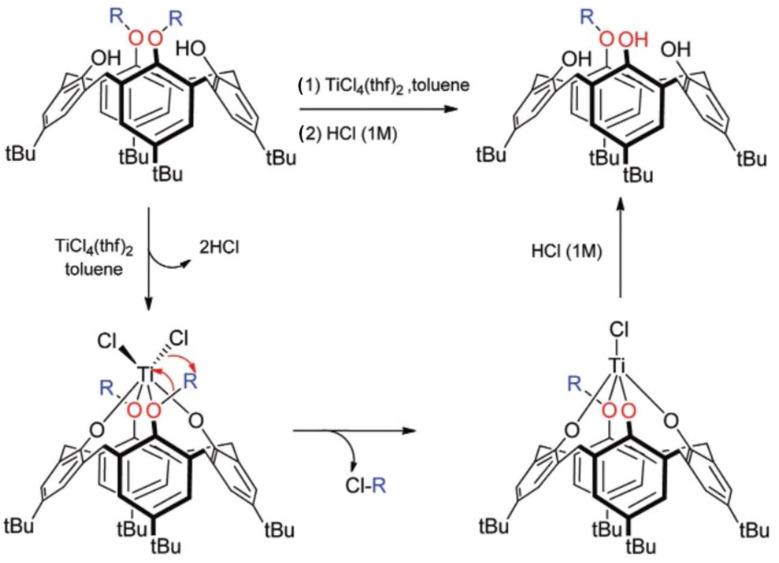
Proposed mechanism for the synthesis of mono-substituted calix[4]arenes from [29].

Other approaches to mono-substitution involved dealkylation from di- or tetra-substituted calix[4]arenes: protecting groups such as iodotrimethylsilane were used on the phenolic OH functionalities [29,30] followed by deprotection of the trimethylsilyl ether. We attempted an alternative approach that avoided protection and de-protection of the OH groups by attempting the dealkylation from a di-substituted derivative using a strong Lewis acid. Both AlCl_3_ [31,32] and TiCl_4_ [33] have been employed in the literature reports. The proposed mechanism for dealkylation using oxophilic TiCl_4_ followed by treatment with aqueous HCl is shown in Scheme 3 [29]. This method worked well in our hands and allowed us to prepare mono-substituted calix[4]arenes.

**Scheme 4:**
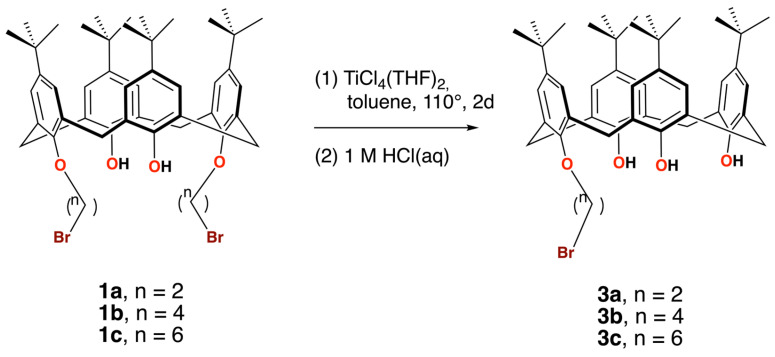
Syntheses of the (ω-bromoalkoxy)t-butylcalix[4]arenes, **3**.

The structures and syntheses of mono(w-bromoalkoxy)t-butylcalix[4]arenes **3a**, **3b**, and **3c** are shown in Scheme 4. Compounds **1** were refluxed in toluene with 1.5 equivalents of TiCl_4_ for two days, during which the reaction mixture turned red, suggesting the formation of TiCl_4_ calixarene complexes, possibly one, or a mixture of both, of the two molecules depicted on the bottom of Scheme 3. The addition of 1 M HCl turned the mixture orange, and colorless compounds **3a**–**3c** were obtained pure via column chromatography. During the syntheses of compound **3** it was observed that the ease of dealkylation to give compounds **3** from **1** increases as the length of the alkyl chain in compound **1** increases. This is also reflected in the increasing yields of the reaction: **3a** (37%) < **3b** (47%) < **3c** (86%).

The lower symmetries of compounds **3** compared to **1** result in more complex ^1^H NMR spectra. The t-Bu protons in **3** exhibit three signals, in a 1:2:1 ratio in their respective spectra. Two signals are seen, in a 2:1 ratio, for the OH groups. Compounds **3** have effective *C*_s_ symmetry in solution and there are three symmetrically distinct aromatic rings. The phenoxy-ring linked to the bromoalkoxy group is bisected by an effective mirror plane (on the ^1^H NMR time scale) and its two protons are chemically equivalent. The same situation holds for the distal phenolic ring, and the two aromatic protons of this ring also appear as a singlet. However while the other two phenolic rings are related by an effective mirror plane, one proton in each ring is closer to the bromoalkoxy subsituent than the other, so the two protons of each ring appear as an AB doublet. Furthermore, based on the coupling constants seen for the two distinct Ar–CH_2_–Ar groups, the calix[4]arenes in all compounds **3** (as seen for compounds **1**) adopt a cone conformation.

### 2.4. Synthesis of Mono- and Bis-ω-alkoxyimidazolium-Substituted Calix[4]arenes

**Scheme 5:**
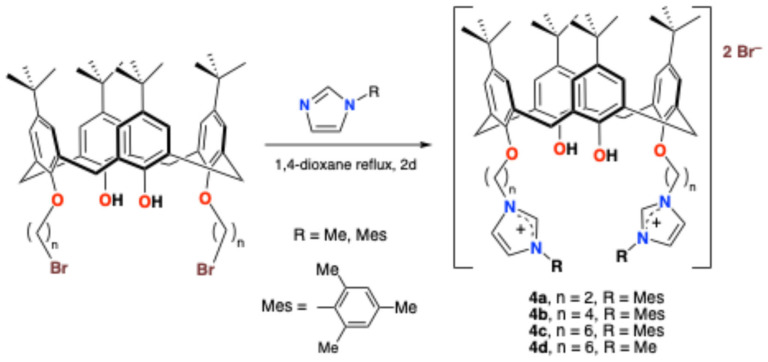
Syntheses of the *bis*-imidazolium compounds **4** (**4a** and **4b** [13]).

The dibromo-compounds **1a** and **1b** were previously reported by us to react with N-substituted imidazoles to afford imidazolium salts **4a** and **4b** (Scheme 5) that are tethered via an alkoxy-chain to opposite ends of the calix[4]arene at the 25 and 27-positions [13]. We extended this synthesis to form **4c** and **4d** from **1c**, which contain –O(CH_2_)_6_– linkages between the imidazolium group and the calixarene.

**Scheme 6:**
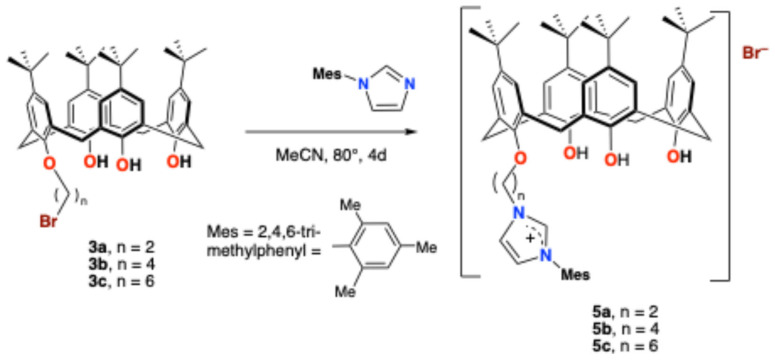
Syntheses of the *mono*-imidazolium compounds.

*Mono*(w-alkoxybromo)t-butylcalix[4]arenes **3** are transformed into *mono*(w-alkoxy-N-mesityl-imidazolium)t-butylcalix[4]arene species **5a**–**5c** by refluxing compounds **3** for 2 days in acetonitile with N-(2,4,6-trimethylphenyl)imidazole (Scheme 6). Compounds **5** are analogs of **4** but have just one imidazolium group tethered to the calix[4]arene skeleton. They were all fully characterized by ^1^H, ^13^C NMR, IR, mass spectroscopy, and elemental analysis, and their melting points were also determined. All NMR data again suggest that the cone-conformation is adopted for these compounds in solution. The compounds are all quite hydroscopic and it is hard to remove all traces of water.

The ^1^H NMR spectra of these salts exhibit an effective mirror plane in solution as is seen for compounds **3**, and their aromatic protons appear as a two singlets and an AB doublet. Signals seen for the CH_2_Br signals in **3** disappear, and new downfield peaks are seen that correspond to the CH_2_N peaks for the methylene group attached to the imidazole nitrogen atom in **5**, as well as new aromatic signals attributed to the imidazolium and mesitylene protons. While the chemical shifts in most of the hydrogen atoms in these imidazolium salts appear in their normal chemical shift ranges, the chemical shifts in all the O*H* protons and frequently the imidazolium protons are somewhat variable, and shifts up to ±0.5 ppm are occasionally observed. Concentration-dependent H-bonding in solution may be responsible for this. In addition, the presence of small quantities of water, which is hard to entirely eliminate, may also form H-bonds in solution with these protons. The melting points for these salts (and other similar salts to be discussed shortly) lie in the 160–240 °C range: longer alkoxyalkyl chain imidazolium compounds **5** have lower melting points.

**Figure 2:**
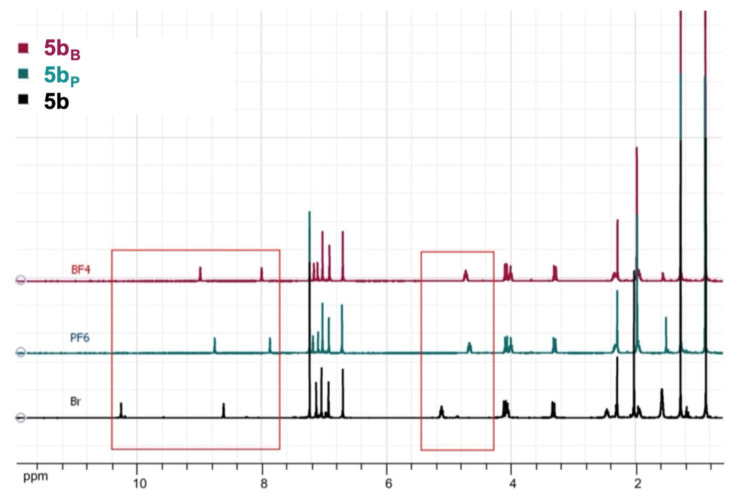
The ^1^H NMR spectra of the cation of **5b** with Br− (**5b**), PF_6_− (**5b_P_**), and BF_4_− (**5b_B_**).

The corresponding PF_6_− or BF_4_− salts of **5b** (**5b_P_** or **5b_B_**) have been prepared by treating **5b** with either KPF_6_ or NaBF_4_, respectively, in anion substitution reactions, in MeOH. The ^1^H NMR spectra of these three compounds (Figure 2) show significant differences in the chemical shifts for the CH_2_N and the acidic imidazolium C–H protons in the spectra of **5b_P_** or **5b_B_** as compared to **5b**, clearly indicating different interactions of the cations of **5b**, **5b_P_**, or **5b_B_** with their respective anions, and thus, anion recognition effects. Further studies of these interactions will be reported later.

**Scheme 7:**
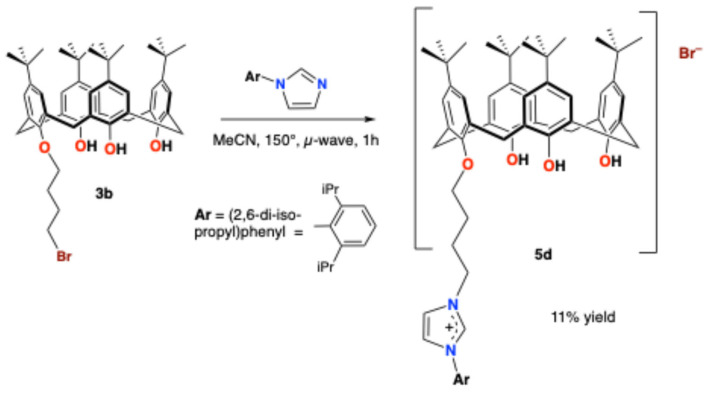
Synthesis of **5d** using microwave irradiation.

The reactions of other imidazoles with **3b** were attempted. The expected analogs of compounds **5b** were indeed formed and detected by ^1^H NMR in reactions with N-methyl-, N-phenyl-, and N-(2,6-diisopropylphenyl)imidazoles. However, in most cases, poor yields combined with significant purification problems led to them not being isolated pure. Only **5d**, formed by reacting N-(2,6-diisopropylphenyl)imidazole with **3b** via microwave synthesis, was obtained pure, in a comparatively low 11% yield as shown in Scheme 7.

**Scheme 8:**
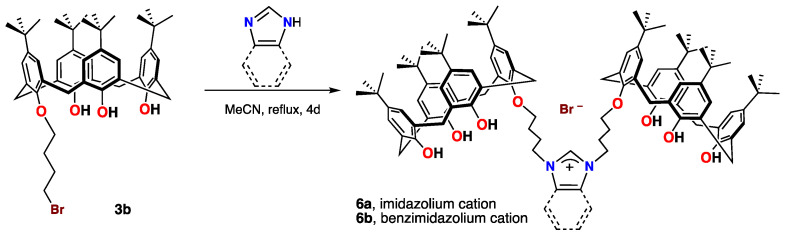
Synthesis of **6a** (X-ray) and **6b** (only observed via mass spectroscopy).

Similar problems were observed in reactions of **3b** with imidazole and benzimidazole, but the imidazole reaction led to small quantities of **6a**, a *(bis*)t-butylcalix[4]arene species linked together by a bridging O–C_4_H_8_–imidazole–C_4_H_8_–O group, as shown in Scheme 8. A similar product **6b** was also detected by mass spectroscopy for the benzimidazole reaction with **3b**.

### 2.5. Single Crystal X-Ray Diffraction of Compound ***6a***

**Figure 3:**
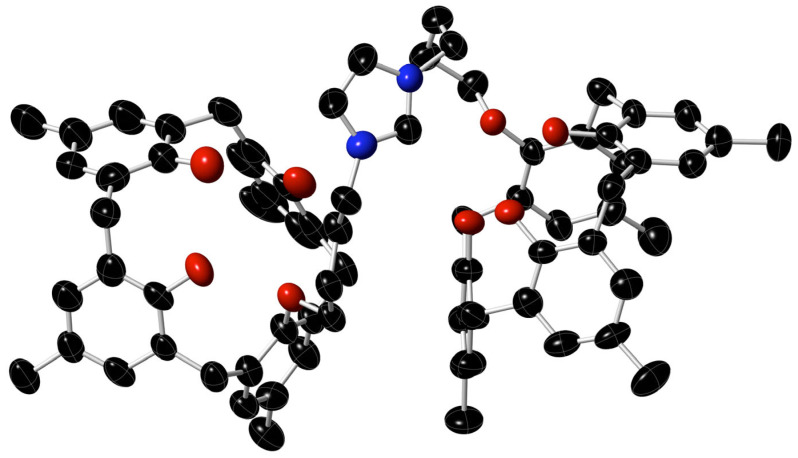
Molecular structure of **6a**, with ellipsoids shown at the 40% level. **Carbon**, **oxygen**, and **nitrogen** atoms are color-coded as shown. Only the central carbon of the eight t-Bu groups is shown., and hydrogen atoms are omitted for clarity.

The structure of **6a** was determined by a single crystal X-ray diffraction study which established the structure shown in Scheme 8. The molecule consists of two t-butylcalix[4]arenes bridged by a O–C_4_H_8_–imidazolium-C_4_H_8_–O- unit. Unfortunately, and as is often the case with calixarenes, this structure exhibits quite significant disorder. Seven of the eight t-Bu groups have each of their Me-groups sitting in two sites. There are large voids in the structure where the bromide anion is presumably sitting, but it could not be accurately located or modeled, and the SQUEEZE program [34] was used to handle this. The molecule crystallizes with two molecules of methanol in the asymmetric unit, which were located. Nevertheless, despite the small crystal size, quality, and the serious disorder problem, we believe the data still allows the molecular geometry to be established as indicated in Scheme 8. Figure 3 depicts the crystallographically determined molecular structure of **6a**.

**Figure 4:**
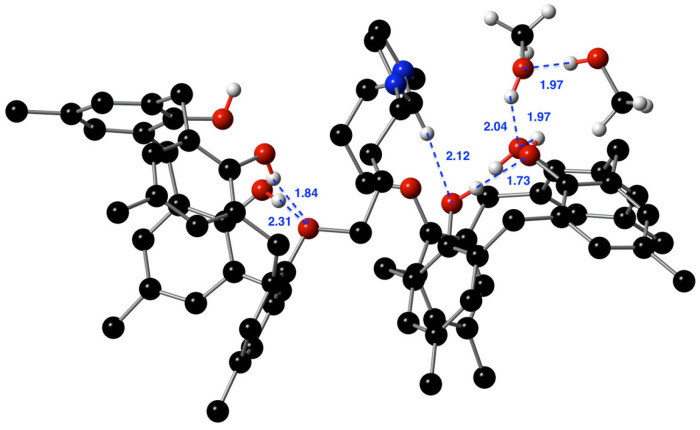
Another view of **6a** showing the H-bond interactions, depicted by dotted blue lines and given in Å. H-bonds between the imidazolium cation and the methanol solvent are also shown. **Carbon**, **oxygen**, and **nitrogen** atoms are color-coded. Most hydrogen atoms that are uninvolved in H-bonding are not shown, and only a single carbon atom is shown for each t-Bu group for clarity.

As observed with the other compounds described here, calix[4]arenes in **6a** adopt cone-conformations. Many of their oxygen atoms are involved in hydrogen-bonding interactions. The 2 OH groups in one calix[4]arene, that flank the oxygen atom that is linked to the C_4_H_8_ chain, have their hydrogen atoms directed towards it and have short O–H --O distances of 1.84 and 2.31 Å, respectively. The other calixarene shows more H-bonding interactions: one of the solvent methanol oxygen atoms shows a O_MeOH_ -- H–O_calix_ distance of 2.04 Å. There are other short O–H–O distances as well as a short imidazolium CH -- OH interaction of 2.12 Å. These are shown in Figure 4.

### 2.6. Reaction of the Mono-Imidazolium Compound ***5a*** with Nickelocene

**Scheme 9:**
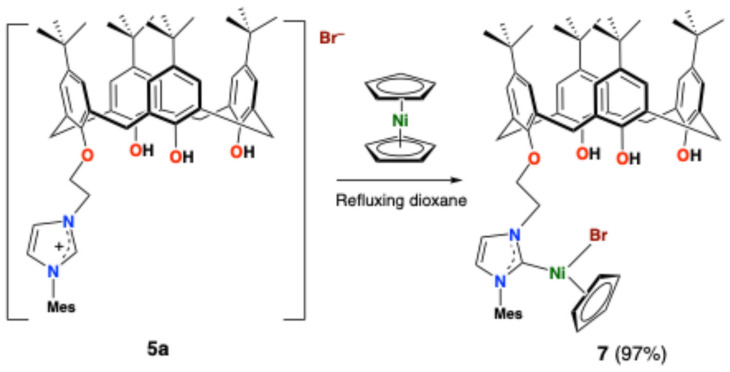
The reaction of **5a** with nickelocene to afford 7, a nickel-NHC complex.

The longer-term goals of the syntheses described in this manuscript are the conversion of the calix[4]arene bound imidazolium salts into nickel N-heterocyclic carbene complexes and a subsequent investigation of the catalytic properties of these molecules. To demonstrate that the synthesis of such species is feasible, we reacted compounds 5a with nickelocene, as shown in Scheme 9.

The reaction proceeded in a similar manner to previously described work [6] with non-calixarene bound imidazolium salts with nickelocene. A mixture of **5a** and nickelocene (carried out under argon as solutions of nickelocene are air-sensitive) led to the green reaction mixture turning red over the 18 h spent in refluxing dioxane, and to the eventual isolation of compound **7** in excellent yield. Unfortunately, good X-ray quality crystals of **7** could not be obtained. However, all other characterization data fully agree with its formulation and structure as shown in Scheme 9.

A signal for the η^5^-C_5_H_5_ protons is seen at δ = 4.78 ppm in the ^1^H NMR. Nickelocene itself is paramagnetic, with two unpaired electrons, and has a reported ^1^H NMR signal at d = −252.9 ppm in 1,2,3,4-tetrafluorobenzene [35,36]. It also shows broad signals in the range of −300 to −200 ppm in the solid-state MAS ^1^H NMR spectrum (these chemical shifts are strongly temperature-dependent [37]).

A corresponding peak for the cyclopentadienyl carbon atoms is observed at d = 92 ppm in the ^13^C NMR. There is also a peak that corresponds to the carbenoid carbon at 163 ppm, in the region characteristic of such Ni-NHC carbon atoms which are found at d = 160 ± 10 ppm typically [38]. None of the other compounds described in this manuscript exhibit a ^13^C NMR chemical shift greater than 150 ppm. The MS and microanalysis data also fully support the proposed formulation: the MS shows a peak with m/e = {M − Br]^+^ with the correct isotopic envelope for a mono-nickel species.

## 3. Materials and Methods

### 3.1. General

All reactions were carried out using Schlenk techniques under an atmosphere of Ar. All chemicals were purchased from the Sigma-Aldrich chemical company (Saint Quentin, France) or Fisher (Illkirch, France). Commercial compounds were used as received. Solvents were purified and dried from traces of water by distillation over Na-benzophenone ketyl (THF, ether, dioxane), Na (toluene) or CaH_2_ (CH_3_CN, CH_2_Cl_2_). Chromatography was carried out on silica gel 60 (63–200 µM). ^1^H and ^13^C NMR spectra were recorded at 298 K either on a FT-Bruker Ultra Shield 300 or on a Bruker Spectrospin 400 spectrometer, operating at 300.13 or 400.14 MHz, respectively, for the ^1^H spectra and at 75.47 or 100.62 MHz for the ^13^C spectra, using CDCl_3_ as the solvent. Chemical shifts (∂) and coupling constants (J) are in ppm and Hz, respectively. Peaks are singlets unless otherwise stated (d = doublet, t = triplet, qn = apparent quintet, sep = septet, m = multiplet). The NMR spectra are shown in the Supplementary Information Tables. IR spectra were collected on a Nicolet 380 FT IR (Thermofisher, Schwerte, Germany) equipped with a diamond SMART ORBIT ATR accessory; the frequencies are recorded in cm^−1^. Microanalytic data and mass spectra were measured by the Analytical and Inorganic Chemistry platform of the Hubert Curien Pluiridisciplinary institute (UMR 7188, CNRS), Strasbourg, France. Mass spectra were obtained on MicroTOF-Q instrument. Melting (or sometimes decomposition) points were registered on fusion capillary Bucchi Melting point M-560 instrument. Chemical products were purchased as received from standard chemical companies (Aldrich). t-Butylcalix[4]arene [39], N,N-bis-2,4,6-trimethylphenyl)imidazolium, and N,N-bis-2,6-diisopropylphenyl)imidazolium chlorides were prepared via literature methods [40,41,42].

### 3.2. Syntheses and Spectroscopic Data of 5,11,17,23-Tetra-t-butyl-26,28-dihydroxy-25,27-bis(2-bromoethoxy)calix[4]arene ***1a***, and ***2***

*para*-t-Butylcalix[4]arene (3.50 g, 5.39 mmol), K_2_CO_3_ (1.90 g, 13.75 mmol), and acetonitrile (50 mL) were placed in a 500 mL round-bottomed flask and stirred for 2 h. Subsequently, 1,2-dibromoethane (4.65 mL, 11.35 mmol) in acetonitrile (150 mL) was added and the mixture was refluxed for 4 d. Heating was then stopped and when the mixture cooled down to ambient temperature, the solvent was removed under vacuum, and the residue was taken up in CH_2_Cl_2_ (150 mL). Excess base was neutralized by addition of 1 M HCl (aq). The mixture was washed with water (2 × 150 mL), and the organic phase was separated and then dried using anhydrous MgSO_4_. Addition of ethyl acetate precipitated excess unreacted p-tert-butylcalix[4]arene, which was removed by filtration. When some CH_2_Cl_2_ was added, a few white crystals of a compound, which was subsequently shown to be **2**, precipitated. The remaining solution was concentrated and then purified by column chromatography on a silica gel column and was eluted using a CH_2_Cl_2_/cyclohexane 50:50 mixture to give **1a** (C_48_H_62_O_4_Br_2_, M = 862.81 g mol^−1^, 1.20 g, 1.39 mmol) as a white powder in 26% yield. ^13^C NMR of **1a** (not reported earlier) [7]: 150.4, 149.2, 147.0, 141.4, 132.3, 127.6, 125.5, 125.0, 75.9, 29.4, 34.0, 33.9, 31.81, 31.08, 31.81, 29.41. ^1^H NMR of **2**: 7.20 (4H, O*H*), 7.07 (8H, Ar-*H*), 6.76 (8H, Ar-*H*), 4.33, and 3.38 (AB doublet, 8H and 8H, Ar-C*H*_2_-Ar, *J* = 13.2), 1.35 (18H, *^t^Bu*), 0.98 (18H, *^t^Bu*).

### 3.3. Synthesis and Spectroscopic Data of 5,11,17,23-Tetra-t-butyl-26,28-dihydroxy-25,27-bis(6-bromohexoxy)calix[4]-arene, ***1c***

t-Butylcalix[4]arene (3.244 g 5.00 mmol) and K_2_CO_3_ (1.54 g, 11.14 mmol) and acetone (50 mL) were placed in a 500 mL round-bottomed flask equipped with an argon inlet and a reflux column. The mixture was stirred for 3 h at ambient temperature. 1,6-dibromohexane (7.58 mL, 19.6 mmol) in acetone (100 mL) was then added, and the mixture was refluxed for 2 d, and then allowed to cool to room temperature. The solvent was removed in vacuo and the residue was dissolved in CH_2_Cl_2_; excess base was neutralized by adding 1 M HCl and the organic layer was separated using a separating funnel, washed with water (2 × 150 mL), and dried over anhydrous MgSO_4_. The filtrate was then concentrated and purified by column chromatography using silica gel and eluted with a mixture of CH_2_Cl_2_–petroleum ether (3:7) and then concentrated. Compound **1c** (**C_56_H_78_Br_2_O_4_**, M = 975.02 g mol^−1^, 1.80 g. 1.85 mmol) was obtained as a white powder in 37% yield. ^1^H NMR: d 7.60 (2H, O*H*), 7.04 (4H, Ar-*H*), 6.81 (4H, Ar-*H*), 4.28, and 3.31 (8H, AB doublet, Ar-C*H*_2_-Ar, *J*_AB_ = 11.2), 3.98 (t, 4H, OC*H*_2_, *J* = 6.5), 3.46 (t, 4H, C*H*_2_-Br, *J* = 6.8), 2.03 (qn, 4H, CH_2_-C*H*_2_-CH_2_-Br, *J* = 5.8), 1.96 (qn, 4H, C*H*_2_-CH_2_-CH_2_-Br, *J* = 3.2), 1.72 (qn, 4H, CH_2_-C*H*_2_-CH_2_-O, *J* = 6.5), 1.62 (qn, 4H, C*H*_2_-CH_2_-CH_2_-O, *J* = 3.5), 1.29 (18H, *t-Bu*), 0.98 (18H, *t-Bu).* ^13^C NMR: 150.9, 150.1, 147.0, 141.6, 132.9, 128.0, 125.7, 125.3, 76.4, 53.3, 34.2, 34.1, 34.0, 33.0, 32.0, 31.9, 31.3, 30.0, 28.2, 25.4.

### 3.4. Synthesis and Spectroscopic Data of 5,11,17,23-Tetra-t-butyl-26,27,28-trihydroxy-25(2-bromoethoxy)calix[4]arene, ***3a***

**1a** (1.00 g, 1.16 mmol) was placed in a 250 mL two-necked round-bottomed flask and TiCl_4_(THF)_2_ (434 mg, 1.30 mmol) was added together with toluene (40 mL). The mixture turned red and was refluxed for 2 d. After cooling, HCl(aq) (100 mL of a 1.0 M solution) were added and the mixture was stirred for 10 h. The orange organic phase was separated and washed with H_2_O (100 mL) and then dried over anhydrous MgSO_4_. The solution was concentrated and then subjected to silica gel column chromatography and eluted with a 50:50 mixture of CH_2_Cl_2_ and petroleum ether. Removal of the solvent led to **3a** (C_46_H_59_BrO_4_, M = 755.86 g mol^−1^, 310 mg, 0.410 mmol, 35%) as a white powder. ^1^H NMR: 10.12 (1H, O*H*), 9.25 (2H, O*H*), 7.06 (2H, Ar-*H*), 7.04 (d, 2H, Ar-*H*, *J* = 2.5), 7.03 (2H, Ar-*H*), 6.97 (d, 2H, Ar-*H*, *J* = 2.3), 4.47 (t, 2H, OC*H*_2_, *J* = 6,), 4.36 (d, 2H, Ar-C*H*_2_-Ar, *J* = 13.2), 4.25 (d, 2H, Ar-*CH*_2_-Ar, *J* = 13.4), 3.98 (t, 2H, C*H*_2_-Br, *J* = 5.9), 3.42 (d, 4H, Ar-C*H*_2_-Ar, *J* = 13.5), 1.20 (9H, *^t^Bu*), 1.19 (18H, *^t^Bu*), 1.16 (9H, *^t^Bu*). ^13^C NMR: 148.6, 148.3, 148.2, 147.5, 143.4, 143.1; 133.2, 128.2, 127.6, 127.4, 126.5, 125.8, 125.6, 125.5, 76.0, 34.3, 34.14, 34.08, 33.1, 32.4, 31.6; 31.3, 29.5.

### 3.5. Synthesis and Spectroscopic Data of 5,11,17,23-Tetra-t-butyl-26,27,28-trihydroxy-25(4-bromobutoxy)calix[4]arene, ***3b***

The dibromo compound **1b** (2.00 g, 2.17 mmol) was placed in a 250 mL round-bottomed flask equipped with an argon inlet and a reflux. TiCl_4_(THF)_2_ (1.50 eq) and toluene (80 mL) were added and the mixture refluxed for 2 d and then cooled to room temperature. HCl aq (100 mL of a 1 M solution) were added, and the mixture was stirred for 10h. The organic phase was then separated, washed twice with water (2 × 100 mL), dried over MgSO_4_, and then filtered and concentrated. The solution was then subjected to column chromatography on silica gel and eluted with a 40:60 mixture of dichloromethane and petroleum ether and was concentrated to give **3b** (**C_48_H_63_BrO_4_**, M = 783.91 g·mol^−1^, 1.47 g, 1.10 mmol) as a white powder in 86% yield. ^1^H NMR: 10.13 (1H, O*H*), 9.52 (2H, O*H*), 7.10 (2H, Ar-*H*), 7.07 (d, 2H, Ar-*H*, *J* = 2.5), 7.00 (2H, Ar-*H*, *J* = 2.3), 4.33 (d, 2H, Ar-C*H*_2_-Ar, *J* = 13.0), 4.28 (d, 2H, Ar-*CH*_2_-Ar, *J* = 13.8), 4.17 (t, 2H, C*H*_2_-O, *J* = 6.1), 3.65 (t, 2H, C*H*_2_-Br, *J* = 6.1,), 3.46 (d, 2H, Ar-*CH*_2_-Ar, *J* = 13.8), 3.44 (d, 2H, Ar-C*H*_2_-Ar, *J* = 13.0), 2.38 (qn, C*H*_2_-CH_2_-O, 2H), 2.27 (qn, C*H*_2_-CH_2_-Br, 2H), 1.24 (9H, *^t^Bu*), 1.22 (18H, *^t^Bu*), 1.20 (9H, *^t^Bu*). ^13^C NMR: 149.3, 148.7, 148.4. 147.9, 143.7, 143.3, 133.6, 128.4, 128.1, 127.8, 126.6, 126.0, 125.8, 76.2, 34.40, 34.25, 34.0, 33.6, 33.1, 32.4, 31.63, 31.62, 31.4, 29.3, 28.6.

### 3.6. Synthesis and Spectroscopic Data of 5,11,17,23-Tetra-tert-butyl-26,27,28-trihydroxy-25(6-bromohexoxy)calix[4]arene, ***3c***

**1c** (1.50 g, 1.54 mmol), TiCl_4_(THF)_2_ (1.5 eq), and toluene (40 mL) were placed in a 250 mL round-bottomed flask equipped with a reflux condenser and an argon inlet, and the mixture was refluxed for 2d. After being allowed to cool to room temperature, HCl aq (100 mL of a 1.0 M solution) were added and the mixture was stirred rapidly for 10 h. The organic phase was separated and washed with water (2 × 100 mL), dried over anhydrous MgSO_4_, and the solution was then concentrated and subjected to column chromatography on silica gel. Elution with a dichloromethane–petroleum ether (40:60), followed by removal of most of the solvent, led to precipitation of **3c** as a white powder (C_50_H_67_BrO_4_, M = 811.97 g mol^−1^, 0.90 g, 1.1 mmol). ^1^H NMR: 10.16 (1H, O*H*), 9.57 (2H, O*H*), 7.07 (2H, Ar–*H*), 7.04 (d, 2H, *J* = 2.4, Ar-*H*), 7.03 (2H, Ar-*H*), 6.97 (d, 2H, Ar-*H, J* = 2.4), 4.33 (d, 2H, Ar-C*H*_2_-Ar, *J* = 12.9), 4.26 (d, 2H, Ar-C*H*_2_-Ar, *J* = 13.4), 4.12 (t, 2H, C*H*_2_-O, *J* = 7), 3.49 (t, 2H, C*H*_2_-Br*, J* = 6.7), 3.44 (d, 2H, Ar-C*H*_2_-Ar, *J* = 5.7), 3.41 (d, 2H, Ar-C*H*_2_-Ar, *J* = 7.2), 2.16 (qn, 2H, CH_2_-C*H*_2_-CH_2_-O, *J* = 7.3), 1.99 (qn, 2H, CH_2_-*CH*_2_-CH_2_-Br, *J* = 7.1), 1.85 (qn, 2H, C*H*_2_-CH_2_-CH_2_-O, *J* = 6.8), 1.69 (qn, 2H, C*H*_2_-CH_2_-CH_2_-Br, *J* = 8.3), 1.20 (9H, *tBu*), 1.19 (18H, *tBu*), 1.17 (9H, *tBu*). ^13^C NMR: 149.5, 148.6, 148.3, 143.8, 143.3, 133.7, 128.5, 128.3, 127.8, 126.6, 126.0, 125.9, 125.8, 76.9, 34.5, 34.2, 34.14, 34.09, 33.2, 32.9, 32.5, 31.7, 31.1, 29.9, 28.2, 25.3. IR: 3295, 3049, 2953, 2866, 1600, 1483, 1455, 1361, 1297, 1201, 1183, 982, 871, 781. ESI-HRMS: *m*/*z* for C_50_H_67_BrO_4_K^+^, 849.3870 (calc. 849.3859). CHN Microanalysis for C_50_H_67_BrO_4_.-0.5H_2_O: Calc.(expt.), C, 73.15 (73.06); H, 8.35 (8.38); N, 0.00 (<0.1).

### 3.7. Synthesis and Spectroscopic Data of 5,11,17,23-Tetra-t-butyl-26,28-dihydroxy-25,27-bis(6-N-(2, 4, 6-trimethylphenyl)imidazoliumhexoxy)calix[4]arene Dibromide, ***4c***

**1c** (0.30 g, 0.3 mmol) were placed in a 25 mL round-bottomed flask equipped with a reflux condenser, and 0.34 g (1.82 mmol) of 2,4,6-trimethylphenyl-imidazole (340 mg, 1.82 mmol) was added followed by dioxane (5 mL) The mixture was heated at 80 °C for 2 days, and then was allowed to cool to room temperature and evaporated to dryness. The residue was dissolved in CH_2_Cl_2_ (1 mL), and Et_2_O (10 mL) and pentane (10 mL) were added, and the mixture was stirred for 1 h. The insoluble precipitate that formed was filtered and wash with Et_2_O (2 × 30 mL) to yield **4c** (C_80_H_106_Br_2_N_4_O_4_, M = 1347.53 g·mol^−1^, 250 mg, 0.18 mmol, 62%) as a beige-colored powder. Melting point: 225 °C. ^1^H NMR: 10.26 (2H, N-C*H*-N), 8.37 (2H, C*H*-N), 7.30 (2H, C*H*-N), 7.11 (2H, O*H*), 7.01 (4H, Ar-*H*), 6.94 (4H, Ar-*H*), 6.72 (4H, Ar-*H*), 4.87 (t, 4H, C*H*_2_-N, *J* = 7.3), 4.16 (d, 4H, Ar-C*H*_2_-Ar, *J* = 13.1), 3.89 (t, 4H, O*CH*_2_, *J* = 5.9), 3.26 (d, 4H, Ar-*CH*_2_*-*Ar, *J* = 13.0), 2.30 (6H, Ar-C*H_3_*), 2.17 (qn, 4H, CH_2_-C*H*_2_-CH_2_-N, *J* = 7.3), 2.04 (12H, Ar-C*H_3_*), 1.89 (qn, 4H, C*H*_2_-CH_2_-CH_2_-N, *J* = 5.1) 1.78 (qn, 4H, CH_2_-C*H*_2_-CH_2_-O, *J* = 7.5 Hz), 1.56 (qn, 4H, C*H*_2_-CH_2_-CH_2_-O, *J* = 6.9 Hz), 1.26 (18H, *^t^Bu*), 0.91 (18H, *^t^Bu*). ^13^C NMR: 150.3, 149.8, 147.0, 141.9, 141.2, 137.7, 134.1, 132.3, 130.8, 129.9, 128.0, 125.5, 125.2, 124.2, 122.9, 76.3, 49.8, 33.93, 33.86, 31.7, 31.6, 31.0, 30.4, 29.9, 25.7, 25.2, 21.1. 17.7. IR: 3381; 3131; 3038; 2951; 2863; 1607; 1564; 1545; 1484; 1461; 1361; 1299; 1200; 1124; 1000; 870; 756; 670; 635. ESI-HRMS: *m*/*z* for C_80_H_106_Br_2_N_4_O_4_ ([M − Br]^+^: calc. 593.4101, expt., 593.408. CHN Microanalysis for C_80_H_106_Br_2_N_4_O_4_.0.5 H_2_O: Calc.(expt.), C, 70.83 (70.80); H, 7.95 (7.90); N, 4.13 (4.46).

### 3.8. Synthesis and Spectroscopic Data of, 5,11,17,23-Tetra-t-butyl-26,28-dihydroxy-25,27-bis (6-N-(1-methylimidazolium)hexoxy)calix[4]arene Dibromide, ***4d***

**3c** (200 mg, 0.20 mmol), 1-methylimidazole (0.15 mL, 1.84 mmol), and dioxane (4 mL) were placed in a 25 mL round-bottomed flask equipped with a reflux condenser, and the mixture was heated to 80° for 2 d. After cooling to ambient temperature, the solvent was removed in vacuo. The residue was dissolved in CH_2_Cl_2_ (1 mL) and diethylether (15 mL) was added, and the mixture was stirred for 1 h. The off-white precipitate that formed was filtered, washed with diethylether (2 × 30 mL), and dried to yield **4d** (C_64_H_90_Br_2_N_4_O_4_, M = 1139.23 g mol^−1^, 127 mg, 0.111 mmol, 54%) as a light-yellow powder. Melting point: 214 °C. ^1^H NMR: 0.24 (s, 2H, N-CH-N), 7.67 (s, 2H, CH-N), 7.40 (s, 2H, CH-N), 7.33 (s, 2H, OH) 7.04 (s, 4H, H-Ar), 6.73 (s, 4H, H-Ar) 4.50 (t, 4H, *J* = 7 Hz, C*H*_2_-N) 4.18 (d, 4H, *J* = 13.1, Ar-C*H*_2_-Ar), 4.04 (6H, N-C*H_3_*); 3.89 (t, 4H, OC*H*_2_, *J* = 6.3,) 3.29 (d, 4H, Ar-C*H*_2_-Ar, *J* = 13.1), 2.04 (qn, 4H, C*H*_2_-CH_2_-CH_2_-N, *J* = 7.2), 1.86 (qn, 4H, *J* = 6.5, CH_2_-CH_2_-CH_2_-N) 1.72 (qn, 4H, C*H*_2_-CH_2_-CH_2_-O, *J* = 7.7) 1.50 (qn, 4H, C*H*_2_-CH_2_-CH_2_-O, *J* = 6.2), 1.27 (18H, *^t^Bu*), 0.91 (18H,*^t^Bu*). ^13^C NMR: 150.4, 149.8, 147.0, 142.0; 137.4, 132.0, 125.5, 125.3, 123.3, 122.7, 76.1, 53.4, 49.7, 36.7, 33.94, 33.89, 33.89, 31.7, 31.0, 30.1, 29.7, 25.7, 25.1. IR: 3369, 3145, 3048, 2952, 2863, 1570, 1484, 1462, 1361, 1297, 1196, 1166, 1124, 998, 871, 733, 621. ESI-HRMS: m/e for C_64_H_90_Br_2_N_4_O_4_: [M − Br]^+^ 1057.6150 (calc. 1057.6140).

### 3.9. Synthesis and Spectroscopic Data of 5,11,17,23-Tetra-t-butyl-26,27,28-trihydroxy-25-2-(N-2,4,6-trimethyl-phenyl)imidazolium)ethoxycalix[4]arene Bromide, ***5a***

**3a** (300 mg, 0.40 mmol), 2,4,6-trimethylphenylimidazole (220 mg, 1.18 mmol), and acetonitrile (4 mL) were added to a 25 mL round-bottomed flask equipped with an argon inlet and a reflux condenser. The mixture was refluxed for 2 d, and then the solvent was removed. Pentane (15 mL) and diethylether (3 mL) were added and the mixture was stirred for 1 h. The white precipitate was filtered and washed with pentane (2 × 30 mL) to give **5a** (C_58_H_73_BrN_2_O_4_, M = 942.11 g mol^−1^, 210 mg, 0.220 mmol, 56%) as a light-beige powder. Melting point: 243 °C. ^1^H NMR: 10.73 (H, N-CH-*N*), 9.87 (H, O*H*), 9.04 (2H, O*H*), 8.49 (H, C*H*-N), 7.26 (H, C*H*-N), 7.06 (d, 2H, Ar-*H*, *J* = 2.3), 7.02 (2H, Ar-*H*), 6.99 (2H, Ar-*H*), 6.97 (d, 2H, Ar-*H*, *J* = 2.2), 5.48 (t, 2H, C*H*_2_-N, *J* = 3.6), 4.70 (t, *J* = 3.6, OC*H*_2_, 2H), 4.11 (d, 2H, Ar-C*H*_2_-Ar, *J* = 2.4), 4.07 (d, 2H, Ar-C*H*_2_-Ar, *J* = 1.4), 3.44 (d, 2H, Ar-C*H*_2_-Ar, *J* = 6.6), 3.40 (d, 2H, Ar-C*H*_2_-Ar, *J* = 7.4), 2.32 (3H, Ar-C*H_3_*), 2.10 (6H, Ar-C*H_3_*), 1.19 (18H, *^t^Bu*), 1.18 (9H, *^t^Bu*), 1.13 (9H, *^t^Bu*). ^13^C NMR: 149.1, 148.5, 148.0, 146.8, 144.3, 143.9, 141.5, 138.3, 134.3, 132.5, 130.8, 130.0, 128.2, 127.7, 126.9, 126.8, 125.9, 125.0, 122.9, 76.2, 51.1, 34.3, 34.1, 34.0, 32.9, 32.2, 31.5, 31.4, 31.1, 21.1, 17.7. IR: 3261, 3049, 2954, 2867, 1598, 1557, 1543, 1483, 1454, 1361, 1297, 1255, 1201, 1163, 1126, 1048, 910, 873, 781, 673. ESI-HRMS: *m*/*z* for C_58_H_73_BrN_2_O_4_: (M – Br)^+^; calc. 861.5570, expt. 861.5600. Microanalysis for C_50_H_67_BrO_4_.0.5H_2_O: Calc. (expt.) C, 72.56 (72.35); H, 7.87 (7.78); N, 2.92 (3.03).

### 3.10. Synthesis and Spectroscopic Data of 5,11,17,23-Tetra-t-butyl-26,27,28-trihydroxy-25-4-N-(2,4,6-trimethylphenyl)imidazolium-butoxycalix[4]arene, ***5b***

**3b** (400 mg, 0.510 mmol), 2,4,6-trimethylphenylimidazole (285 mg, 1.53 mmol), and acetonitrile (5 mL) were placed in a 25 mL round-bottomed flask equipped with an argon inlet and a reflux condenser, and the mixture was refluxed for 2 d, cooled, and evaporated to dryness. Pentane (30 mL) was added, and the suspension was stirred for 2 h and then filtered and washed with pentane (2 × 30 mL), and dried to give **5b** (C_60_H_77_BrN_2_O_4_, M = 970.17 g mol^−1^, 390 mg 0.40 mmol, 79%) as a light-yellow powder. Melting point: 228 °C. ^1^H NMR: 10.68 (1H, N-C*H*-N), 10.32 (1H, O*H*), 9.58 (2H, O*H*), 8.06 (1H, C*H*-N), 7.17 (1H, C*H*-N), 7.07 (d, 2H, Ar-*H*, *J* = 2.0), 7.06 (2H, Ar-*H*), 7.04 (2H, Ar-*H*), 6.98 (2H, Ar-*H*), 6.97 (d, 2H, Ar-*H*, *J* = 2.2), 5.11 (t, 2H, C*H*_2_-N, *J* = 7.5), 4.23 (d, 2H, Ar-C*H*_2_-Ar, *J* = 13.0), 4.15 (d, 2H, Ar-C*H*_2_-Ar, *J* = 13.2), 4.14 (t, 2H, OC*H*_2_, *J* = 6.1), 3.43 (d, 4H, Ar-C*H*_2_-Ar, *J* = 13.0), 2.71 (qn, 2H, C*H*_2_-CH_2_-N, *J* = 7.4), 2.32 (3H, Ar-C*H_3_*), 2.31 (qn, 2H, C*H*_2_-CH_2_-O, *J* = 7.1), 2.09 (6H, Ar-C*H_3_*), 1.20 (9H, *^t^Bu*), 1.19 (18H, *^t^Bu*), 1.16 (9H, *^t^Bu*). ^13^C NMR: 148.8, 148.6, 148.0, 147.3, 144.3, 143.7, 141.4, 138.4, 134.3, 133.2, 130.7, 129.9, 128.3, 128.2, 127.3, 126.6, 125.9, 125.8, 123.3, 122.8, 49.7, 34.3, 34.1, 34.0, 33.1, 32.1, 31.49, 31.46, 31.2, 27.3, 26.5, 21.1, 17.7.

IR: 3241, 3150, 3019, 2957, 2869, 1597, 1563, 1549, 1483, 1461, 1392, 1361, 1288, 1202, 1068, 1005, 871, 781, 670. ESI-HRMS: *m*/*z* for C_60_H_77_BrN_2_O_4_: (M − Br]^+^ calc. 889.5877; expt. 889.5851. CHN Microanalysis for C_60_H_77_BrN_2_O_4_.0.5MeCN: Calc.(expt.), C, 73.95 (73.90); H, 7.99 (8.02); N, 3.53 (3.39).

### 3.11. Synthesis of the Hexafluorophosphate and Tetrafluoroborate Salts of ***5b***, 5,11,17,23-Tetra-t-butyl-26,27,28-trihydroxy-25-(4-N-(2,4,6-trimethylphenyl)imidazoliumbutoxy)-calix[4]arene Hexafluorophosphate (***5b_P_***) and Tetrafluoroborate (***5b_B_***)

**5b**, (100 mg, 0.100 mmol) and methanol (30 mL) were placed in a 50 mL round-bottomed flask. KPF_6_ (230 mg, 1.20 mmol) or NaBF_4_ (136 mg, 1.20 mmol) were added together with water (3 mL) and the mixture stirred for 18 h at ambient temperature. The solvent was then removed, and the residue was washed with water (15 mL). Dichloromethane (25 mL) was then added, and the mixture was vigorously shaken. The organic phase was separated, dried over MgSO_4_, filtered, and the solvent was then concentrated and placed on a silica gel column. Elution using a 10:1 mixture of CH_2_Cl_2_/EtOH afforded **5b_P_** (C_60_H_77_F_6_N_2_O_4_P, M = 1035.55 g mol ^−1^, 60 mg, 0.058 mmol, 58%) or **5b_B_** (C_60_H_77_BF_4_N_2_O_4_, M = 977.09 g mol^−1^, 48 mg, 0.049 mmol, 49%) as pale-yellow powders.

**Data for 5b_P_**: Melting point, 174 °C. ^1^H NMR: δ 10.31 (1H, O*H*), 9.58 (2H, O*H*), 8.97 (1H, N-C*H*-N), 7.98 (1H, C*H*-N), 7.22 (1H, C*H*-N), 7.08 (d, 2H, Ar-*H*, *J* = 2.1), 7.07 (2H, Ar-*H*), 7.03 (2H, Ar–*H*), 6.99 (2H, Ar-*H*), 6.97 (d, 2H, Ar-*H*, *J* = 2.2), 4.74 (t, 2H, C*H*_2_-N, *J* = 7.9), 4.23 (d, 2H, Ar-C*H*_2_-Ar, *J* = 13.1), 4.14 (t, 2H, OC*H*_2_, *J* = 6.3), 4.12 (d, 2H, Ar-C*H*_2_-Ar, *J* = 13.6), 3.45 (d, 2H, Ar-C*H*_2_-Ar, *J* = 13.8), 3.41 (d, 2H, Ar-C*H*_2_-Ar, *J* = 13.4), 2.67 (qn, 2H, C*H*_2_-CH_2_-N, *J* = 7.5), 2.32 (3H, Ar-C*H_3_*), 2.23 (qn, 2H, C*H*_2_-CH_2_-O, *J* = 6.4), 2.05 (6H, Ar-C*H_3_*), 1.20 (9H, ^t^Bu), 1.19 (18H, ^t^Bu), 1.16 (9H, *^t^Bu*). ^13^C NMR: 148.7, 148.0, 147.3, 144.3, 143.8, 141.5, 136.8, 134.4, 133.2, 129.9, 128.2, 127.4, 126.7, 125.9, 125.8, 123.9, 123.2, 75.5, 50.0, 34.3, 34.0, 33.1, 32.1, 31.5, 31.2, 27.0, 26.5, 21.1, 17.3. ^19^F NMR (376 MHz): −72.36 (d, *J* = 712.4, P*F*_6_). ^31^P NMR (161 MHz): −144.32 (septet, *J* = 712.2, *P*F_6_).

**Data for 5b_B_**: Melting point: 213 °C. ^1^H NMR: 10.31 (1H, O*H*), 9.58 (2H, O*H*), 9.39 (1H, N-C*H*-N), 7.98 (1H, C*H*-N), 7.22 (1H, C*H*-N), 7.08 (d, Ar-*H*, 2H, *J* = 2.1), 7.07 (2H, Ar-*H*), 7.03 (2H, Ar-*H*), 6.99 (2H, Ar-*H*), 6.97 (d, 2H, Ar-*H*, *J* = 2.2), 4.88 (t, 2H, C*H*_2_-N, *J* = 7.9), 4.23 (AB d, 2H, Ar-C*H*_2_-Ar, *J* = 13.1), 4.14 (t, 2H, OC*H*_2_, *J* = 6.3), 4.12 (AB d, 2H, Ar-C*H*_2_-Ar, *J* = 13.6), 3.45 (AB d, 2H, Ar-C*H*_2_-Ar *J* = 13.8), 3.41 (AB d, 2H, Ar-C*H*_2_-Ar *J* = 13.4), 2.67 (qn, 2H, C*H*_2_-CH_2_-N, *J* = 7.5), 2.32 (3H, Ar-C*H_3_*), 2.23 (qn, 2H, C*H*_2_-CH_2_-O, *J* = 6.4), 2.05 (6H, Ar-C*H_3_*), 1.20 (9H, *^t^Bu*), 1.19 (18H, *^t^Bu*), 1.16 (9H, *^t^Bu*). ^13^C NMR: 148.7, 148.0, 147.3, 144.3, 143.8, 141.5, 136.8, 134.4, 133.2, 129.9, 128.2, 127.4, 126.7, 125.9, 125.8, 123.9, 123.2, 75.5, 50.0, 34.3, 34.0, 33.1, 32.1, 31.5, 31.2, 27.0, 26.5, 21.1, 17.3. ^19^F NMR (376 MHz) −151.8 (B*F_4_*). ^11^B NMR (128 MHz) −0.88 (*B*F_4_).

### 3.12. Synthesis and Spectroscopic Data of 5,11,17,23-Tetra-t-butyl-26,27,28-trihydroxy-25(6-(2,4,6-trimethylphenyl)imidazolium-hexoxy)calix[4]arene Bromide, ***5c***

The mono-substituted calixarene **3c** (300 mg, 0.37 mmol) was placed in a 25 mL round-bottomed flask equipped with a reflux condenser and 2,4,6-trimethylphenylimidazole (410 mg, 2.2 mmol) and dichloromethane (5 mL). The mixture was refluxed for 2 days, and after cooling the solution was subjected to column chromatography. Elution with a CH_2_Cl_2_/EtOH mixture (10:1) and concentration of the eluted solution yielded **5c** (C_62_H_81_BrN_2_O_4_, M = 998.22 g·mol^−1^, 110 mg, 0.11 mmol, 30%) as a pale-yellow powder. Melting point, 150 °C. ^1^H NMR: 10.62 (1H, N-C*H*-N), 10.23 (1H, O*H*), 9.56 (2H, O*H*); 7.09 (1H, C*H*-N), 7.06 (2H, Ar-*H*), 7.04 (d, 2H, *J* = 2.5, Ar-*H*), 7.02 (2H, Ar-*H*), 6.97 (2H, Ar-*H*), 6.96 (d, 2H, *J* = 2.5, Ar-H), 4.84 (t, 2H, *J* = 7.3, C*H*_2_-N), 4.28 (d, 2H, *J* = 13.0, Ar-C*H*_2_-Ar), 4.19 (d, 2H, *J* = 13.6, Ar-C*H*_2_-Ar), 4.09 (t, 2H, *J* = 6.3, OC*H*_2_), 3.42 (d, 2H, *J* = 2.2, Ar-C*H*_2_-Ar), 3.38 (d, 2H, *J* = 2.2, Ar-C*H*_2_-Ar), 2.31 (3H, Ar-C*H_3_*), 2.19 (qn, 2H, *J* = 7.5, C*H*_2_-CH_2_-N), 2.12 (qn, 2H, *J* = 7.1, C*H*_2_-CH_2_-O), 2.06 (6H, Ar-C*H_3_*) 1.86 (qn, 2H, *J* = 7.3, C*H*_2_-CH_2_-CH_2_-N), 1.64 (qn, 2H, *J* = 7.7, C*H*_2_-CH_2_-CH_2_-O), 1.20 (9H, ^t^Bu); 1.18 (18H, ^t^Bu); 1.16 (9H, ^t^Bu). ^13^C NMR: 148.9, 148.8, 148.61, 147.3, 143.9, 141.3, 138.3, 134.2, 133.4, 130.7, 129.90, 128.2, 127.5, 126.5, 125.8, 123.2, 122.9, 49.8, 34.4; 34.2, 34.1, 33.2, 32.2, 31.61, 31.57, 31.3, 27.5, 26.8, 21.2, 17.8. IR: 3338, 3047, 2959, 2868, 1596, 1562, 1543, 1483, 1390, 1362, 1298, 1242, 1193, 1122, 1060, 870, 804, 758, 672. ESI-HRMS: *m*/*z* for C_63_H_813_BrN_2_O_4_, (M − Br)^+^: calc. 931.635; expt. 931.630.

### 3.13. Synthesis and Spectroscopic Data of 5,11,17,23-Tetra-t-butyl-26,27,28-trihydroxy-25-(4-(2,6-diisopropylphenyl)imidazolium-butoxy)calix[4]arene Bromide, ***5d***

**3b** (150 mg, 0.191 mmol), 2,6-diisopropylphenylimidazole (131 mg, 0.57 mmol), and acetonitrile (4 mL) were placed in a 5 mL vial, sealed, and heated to 150 °C for 1 h. After cooling, the solvent was removed, the residue was dissolved in a minimum of CH_2_Cl_2_, and the product was precipitated by addition of pentane to give **5d** (C_63_H_83_BrN_2_O_4_, M = 1012.25 g mol^−1^, 20 mg, 0.021 mmol, 11%) as a light-yellow powder. ^1^H NMR: 10.80 (1H, N-C*H*-N), 10.37 (1H, O*H*), 9.61 (2H, O*H*), 8.07 (1H, C*H*-N), 7.52 (t, 1H, *J* = 7.8, Ar-*H*), 7.28 (d, 2H, *J* = 7.8, H-Ar), 7.20 (1H, C*H*-N), 7.07 (d, 2H, *J* = 2.1,Ar-H), 7.04 (2H, Ar-*H*), 7.02 (2H, Ar-*H*), 6.96 (d, 2H, *J* = 2.2, Ar-*H*), 5.20 (t, 2H, *J* = 7.1, C*H*_2_-N), 4.23 (d, 2H, *J* = 13.2, Ar-C*H*_2_-Ar), 4.17 (d, 2H, *J* = 13.6, Ar-C*H*_2_-Ar), 4.15 (t, 2H, *J* = 6.0, OC*H*_2_), 3.46 (d, 2H, *J* = 2.3, Ar-C*H*_2_-Ar), 3.42 (d, 2H, *J* = 2.9, Ar-C*H*_2_-Ar), 2.73 (qn, 2H, *J* = 7.3, C*H*_2_-CH_2_-N), 2.34 (m, 2H, *J* = 6.8, CH(C*H_3_*)_2_), 2.34 (qn, 2H, *J* = 6.8, C*H*_2_-CH_2_-O), 1.23 (d, 6H, *J* = 6.1, CH(C*H_3_*)_2_), 1.20 (9H, *^t^Bu*), 1.19 (18H, *^t^Bu*), 1.16 (9H, *^t^Bu*), 1.06 (d, 6H, *J* = 6.5, CH(C*H_3_*)_2_. ^13^C NMR: 148.8, 148.7, 148.0, 147.3, 145.5, 144.4, 143.8, 139.0, 133.2, 131.9, 130.2, 128.3, 128.2, 127.3, 126.6, 125.9, 125.8, 124.7, 124.6, 124.3, 122.7, 75.8, 49.8, 34.3, 34.1, 34.0, 33.1, 32.1, 31.5, 31.2, 28.8, 28.0, 27.4, 26.6, 26.0, 24.5, 24.0. IR: 3338, 3047, 2959, 2868, 1596, 1562, 1543, 1483, 1390, 1362, 1298, 1242, 1193, 1122, 1060, 870, 804, 758, 672. ESI-HRMS: *m*/*z* for C_63_H_83_N_2_O_4_ (M − Br^+^): 931.635, calc. 931.63.

### 3.14. Synthesis and Spectroscopic Data of 7, Bromo-(η^5^-cyclopentadienyl) {5,11,17,23-Terta-tert-butyl-25-(2-(2,4,6-trimethylphenyl)imidazole-2-ylidiene-ethyloxy)-26,27,28-trihydroxycalix[4]-arene)nickel

**5a** (100.0 mg, 0.106 mmol) and nickelocene (24 mg, 0.127 mmol) were placed in a 25 mL Schlenk tube under argon, and 1,4-dioxane (5 mL) was added. The mixture was refluxed under argon for 18 h and the green solution slowly turned reddish brown. After being allowed to cool to ambient temperature, the solution was filtered through a Celite pad to give a deep red filtrate. The dioxane was concentrated under vacuum, pentane (15 mL) was added, and after cooling in a −20 °C freezer, **7** (C_63_H_78_BrN_2_NiO_4_, M = 1065.92 g·mol^−1^, 110 mg, 0.103 mmol, 97%) was obtained as deep red powder. ^1^H NMR: 9.93 (1H, C*H*N), 9.20 (2H, O*H*), 7.98 (1H, C*H*N); 7.07 (2H, Ar-*H*), 7.06 (d, 2H, Ar-*H, J* = 2.7), 7.02 (2H, Ar-*H*), 6.99 (1H, O*H*), 6.97 (d, 2H, Ar-H, *J* = 2.3), 5.64 (t, 2H, *OCH*_2_ *J* = 7.6), 4.99 (t, 2H, C*H*_2_-N, *J* = 8.8), 4.80 (5H, C_5_*H_5_*), 4.30 and 4.17 (AB d, 4H, Ar-C*H*_2_-Ar, *J* = 12.9), 3.47 and 3.43 (d, 2H, Ar-C*H*_2_-Ar, *J* = 13.5), 2.40 (3H, Ar-C*H_3_*), 1.20 (18H, *^t^Bu*), 1.20 (6H, Ar-C*H_3_*), 1.19 (9H, *^t^Bu*), 1.16 (9H, ^t^*Bu*). ^13^C NMR: 163.6, 149.4, 148.6, 148.4, 147.3, 144.0, 143.4, 139.2, 137.0, 133.0, 129.3, 129.2, 128.3, 127.7, 127.0, 126.8, 125.9, 125.8, 125.7, 125.4, 124.0, 92.0, 77.2, 76.7, 52.8, 34.3, 34.1, 34.0, 33.0, 32.3, 31.5, 31.4, 31.2, 21.2. IR: 3263, 3159, 3029, 3012, 2951, 2869, 1597, 1561, 1549, 1538, 1469, 1482, 1366, 1328, 1219, 1168, 1011, 898, 781, 693. ESI-HRMS: *m*/*z* for C_63_H_78_BrN_2_NiO_4_ ([M − Br]^+^) 984.5321, calc. 984.5315. CHN Microanalysis for C_63_H_78_BrN_2_NiO_4_: Calc.(expt.), C, 70.99 (70.75); H, 7.38 (7.31); N, 2.63 (2.51).

### 3.15. Single Crystal X-Ray Diffraction Studies

X-ray diffraction data for **2** and **6a** were all collected on a Bruker APEX II DUOKappa-CCD diffractometer (Karlsruhe, Germany) equipped with an Oxford Cryosystem liquid N_2_ device, using Mo-Kα radiation (λ = 0.71073 Å) at the University of Strasbourg structural facility. All structures were solved using SHELXS-97 [43,44]. Single crystals of **2** and **6a** were obtained by crystallization at 4 °C from dichloromethane (**2**) or as small crystals from acetonitrile. The crystal–detector distance was 38 mm. The cell parameters were determined (APEX2 software, [45]) from reflections taken from three sets of six frames, each at 10 s exposure. The refinement and all further calculations were carried out using SHELXL-2019 [46]. The H-atoms were included in calculated positions and treated as riding atoms using SHELXL default parameters. The non-H atoms were refined anisotropically, using weighted full-matrix least-squares on F2. A semi-empirical absorption correction was applied using SADABS in APEX2 [39]. For compounds **2** and **6a**, the SQUEEZE instruction in PLATON [32] was applied, and for **6** the residual electron density corresponded to a bromide and 3.5 molecules of methanol.

## 4. Conclusions

New mono- and di-distal imidazolium cations **4** and **5** that are linked to para-t-butylcalix[4]arenes along the outer rim have been synthesized and fully characterized. In addition, the interesting *quasi*-dimeric molecules **2** and **6** were isolated in small quantities. The formation of nickel N-heterocyclic carbene complexes from the *mono-*substituted calix[4]arene imidazolium salts is envisaged, and this is shown to be feasible with the reaction depicted in Scheme 9. Such complexes have the potential to act as Suzuki-Miyaura catalysts and is the subject of current research. There are also indications that some of the imidazolium salts have potential in cation recognition. Future work is planned to investigate this further.

## Data Availability

The original contributions presented in the study are included in the Supplementary Materials. Crystallographic data for compounds **2** and **6a** have been deposited with the Cambridge Crystallographic Data Centre as supplementary publication nos. CCDC 2475852 and 2475853. Copies of the data can be obtained free of charge from the Director, CCDC, 12 Union Road, Cambridge CB2 1EZ, UK (fax: +44-1223-336-033; e-mail: deposit@ccdc.cam.ac.uk; http://www.ccdc.cam.ac.uk).

## Supplementary Materials

The following supporting information can be downloaded at: https://www.mdpi.com/article/10.3390/molecules30193954/s1, Spectroscopic ^1^H and ^13^C NMR spectra for the new compounds, and X-ray data, including the CheckCif files for **2** and **6a**.
