# Supplementary material for: Synthesis of Imidazolium Salts Linked to a t-Butylcalix[4]arene Framework and the Isolation of Interesting By-Products
Source: Molecules. 2025 Oct 1;30(19):3954. doi: 10.3390/molecules30193954 (PMC12526095; doi:10.3390/molecules30193954)
Supplement: Supplementary file 1 [file molecules-30-03954-s001.zip › molecules-3816973-supplementary.pdf]

Supplementary Information

Synthesis of Imidazolium Salts Linked to a t-Butylcalix[4]arene Framework and the Isolation of Interesting By-Products

Michael J. Chetcuti [1,*], Rahma Aroua [1] and Abdelwaheb Hamdi [2]

[1] Organometallic Chemistry Group, LIMA—UMR CNRS 7042, European School of Chemistry, Polymers and Materials (ECPM), Universities of Strasbourg and of Upper Alsace, 25 Rue Becquerel, 67087 Strasbourg, France; rahma.aroua@gmail.com
[2] LR05ES09 Laboratory of Applied Chemistry and Natural Substances Resources and Environment (LACReSNE), Faculty of Sciences of Bizerte, University of Carthage, Bizerte 7021, Tunisia; abdelwaheb.hamdi@istmt.utm.tn
* Correspondence: michael.chetcuti@unistra.fr

Pages 2 – 8 ^{1}H and ^{13}C NMR spectra of compounds

2	**1c**
3	**2**
3	**3a**
4	**3b**
5	**3c**
6	**4c**
7	**4d**
8	**5a**
9	**5b**
10	**5c**
11	**5d**
12	**7**

Page 14 CheckCif for compound **2**

Page 18 CheckCif for compound **6a**

^{1}H NMR spectrum of 1c

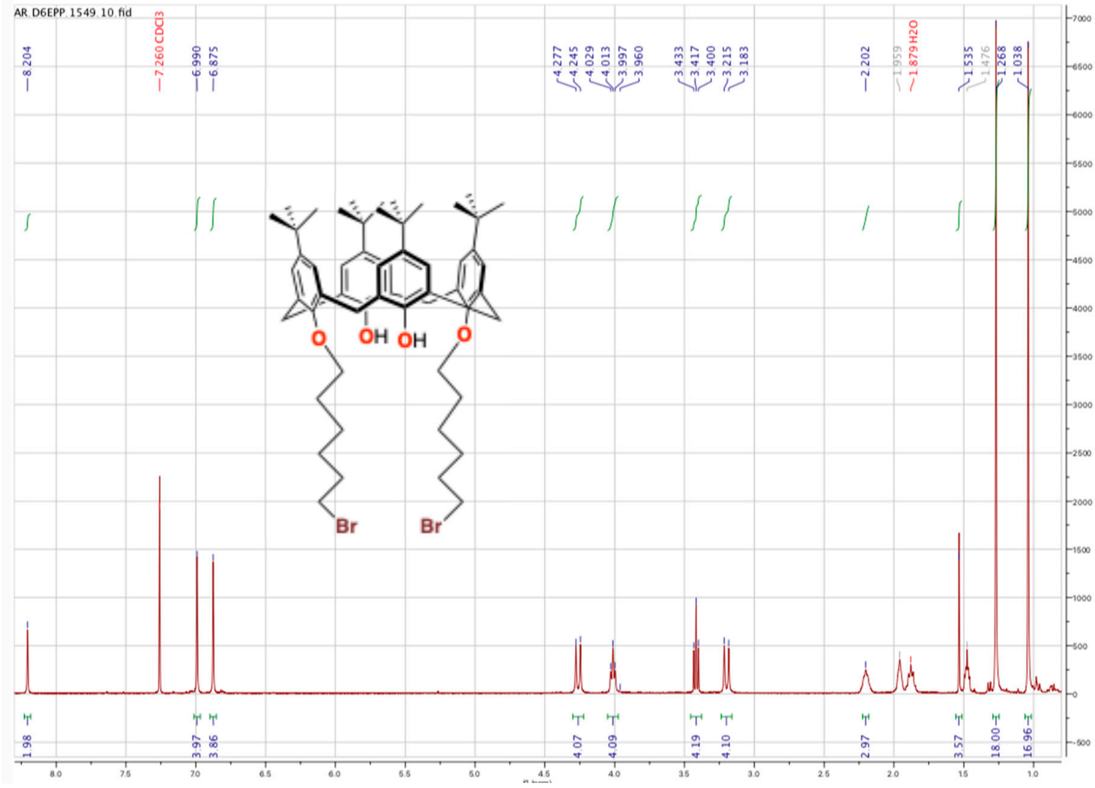

^{13}C NMR spectrum of 1c

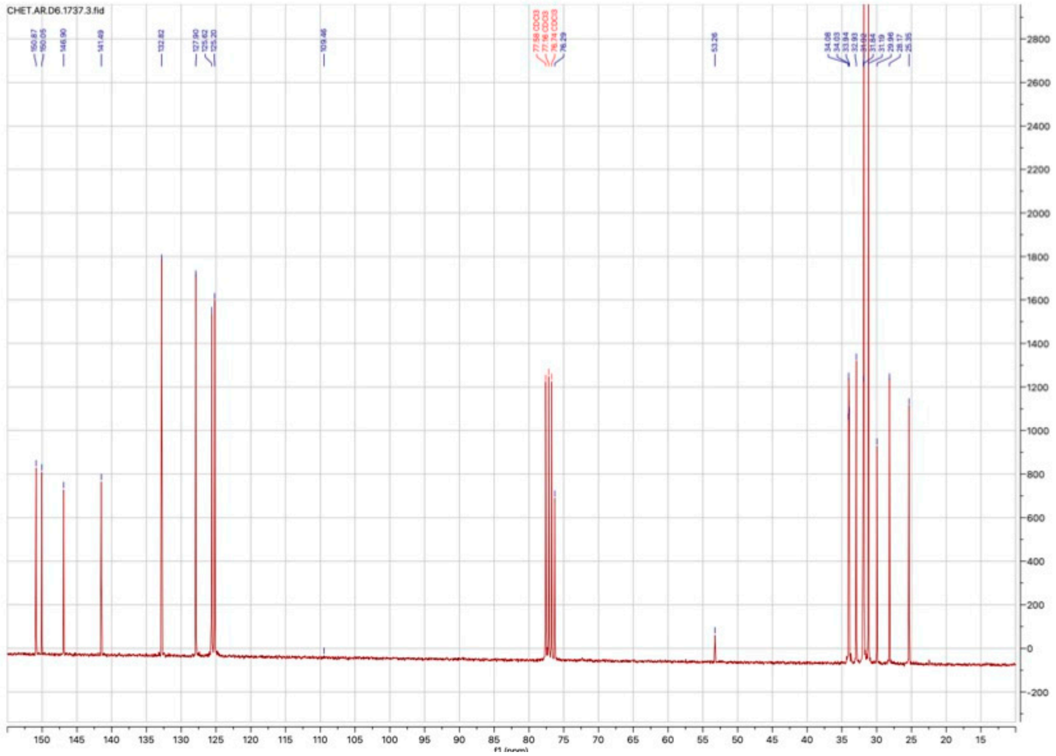

^{1}H NMR spectrum of 2

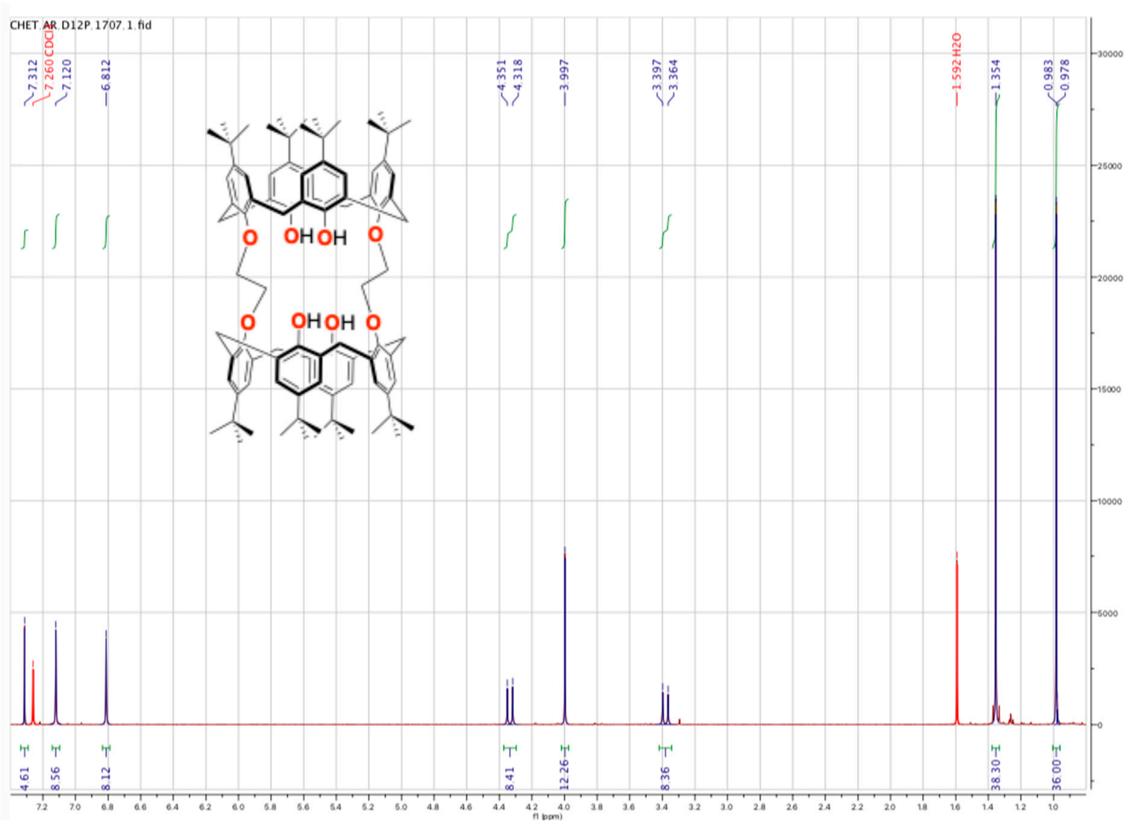

^{1}H NMR spectra of 3a

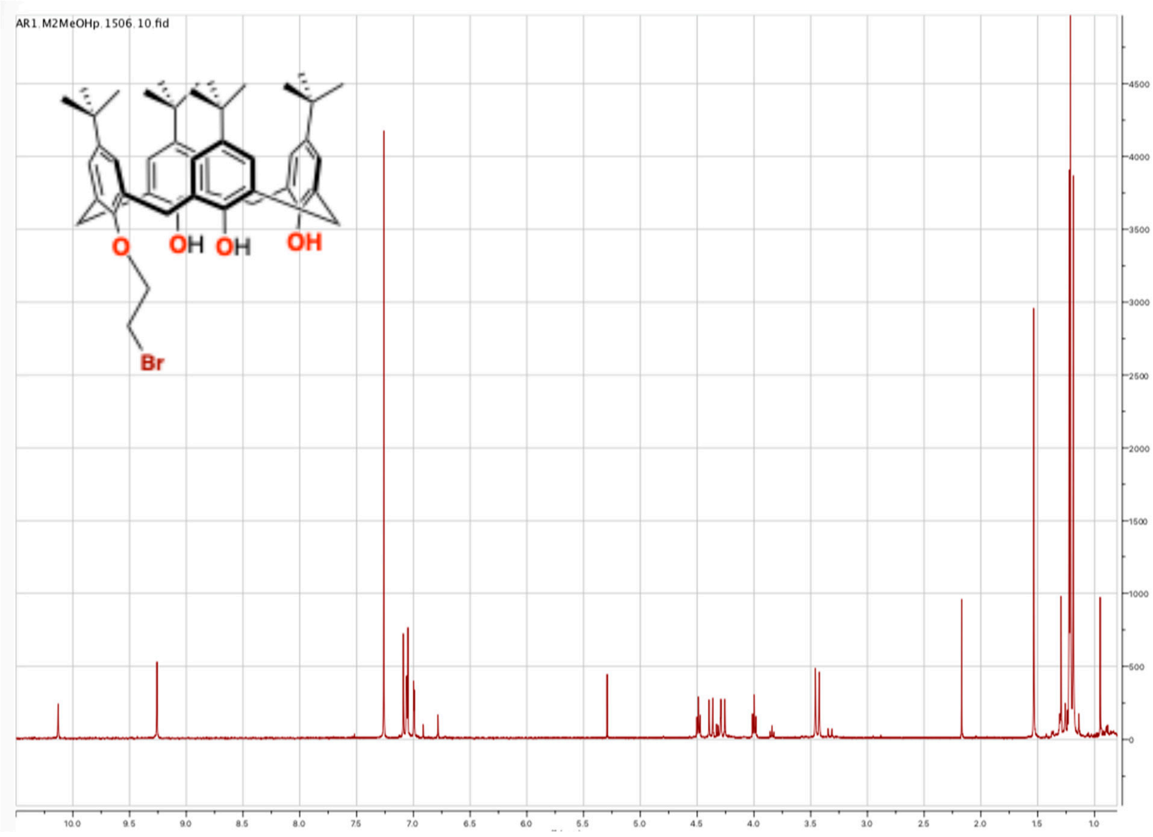

^{13}C NMR spectrum of 3a

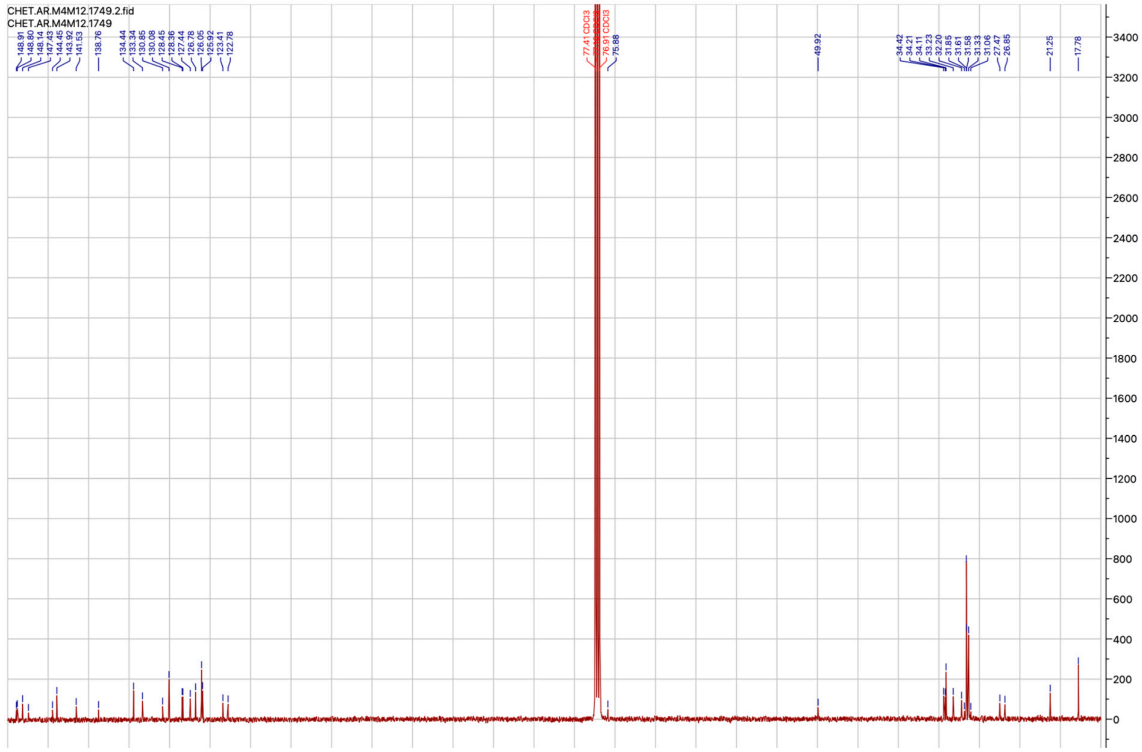

^{1}H NMR spectra of 3b

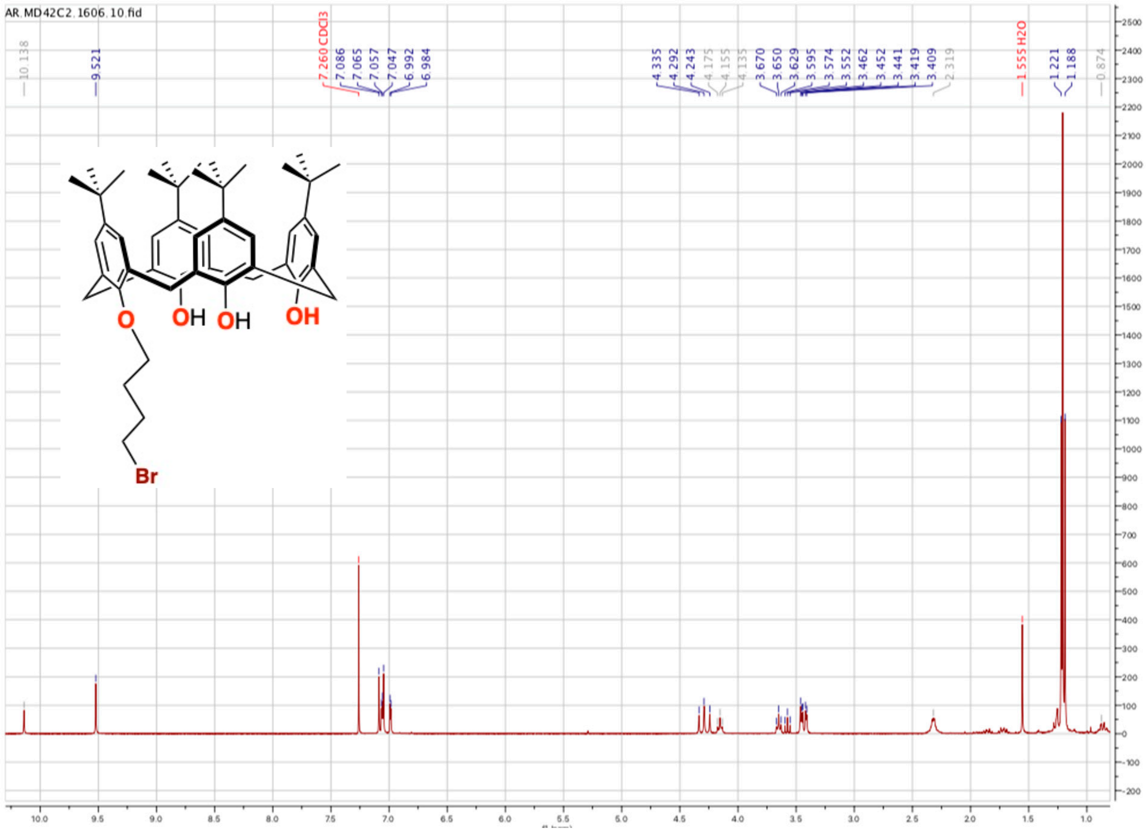

^{13}C NMR spectrum of 3b

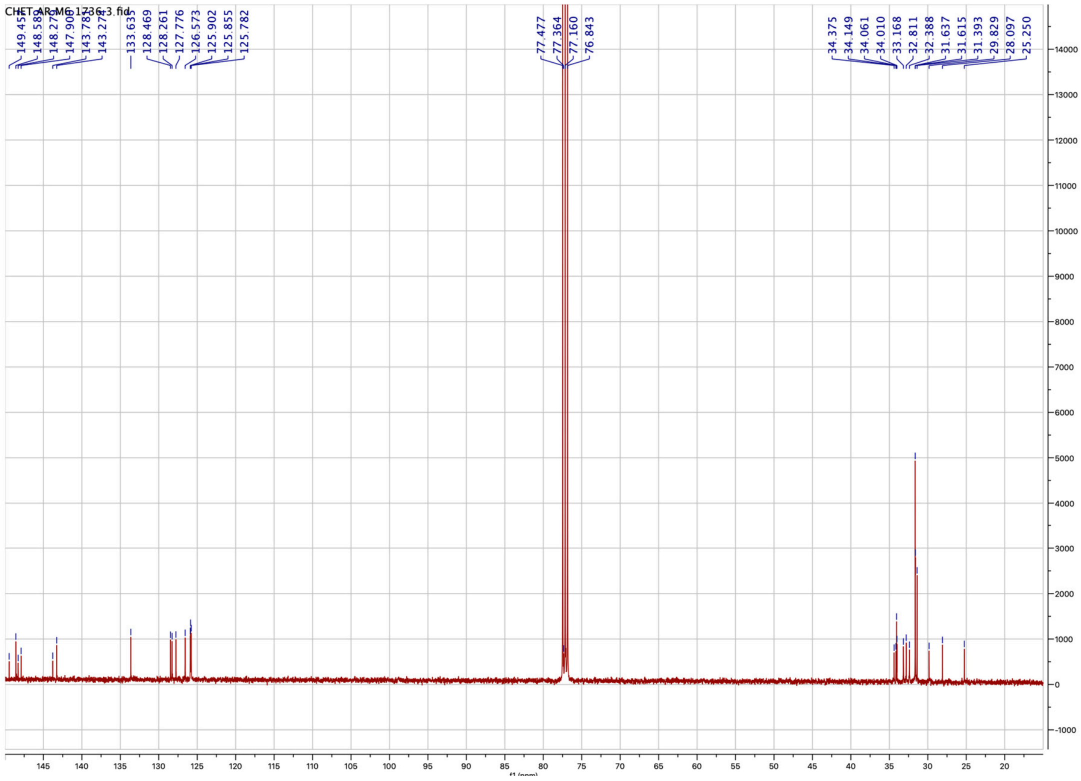

^1H NMR spectrum of 3c

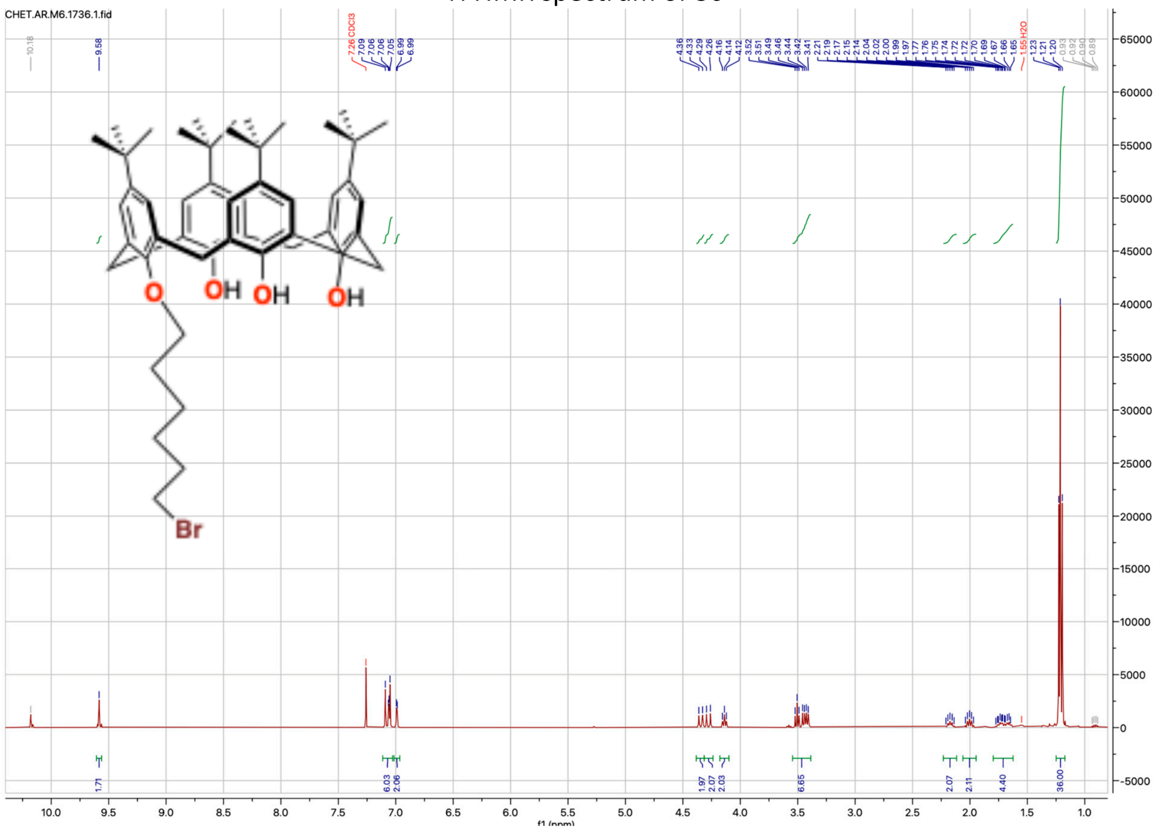

^{13}C NMR spectrum of 3c

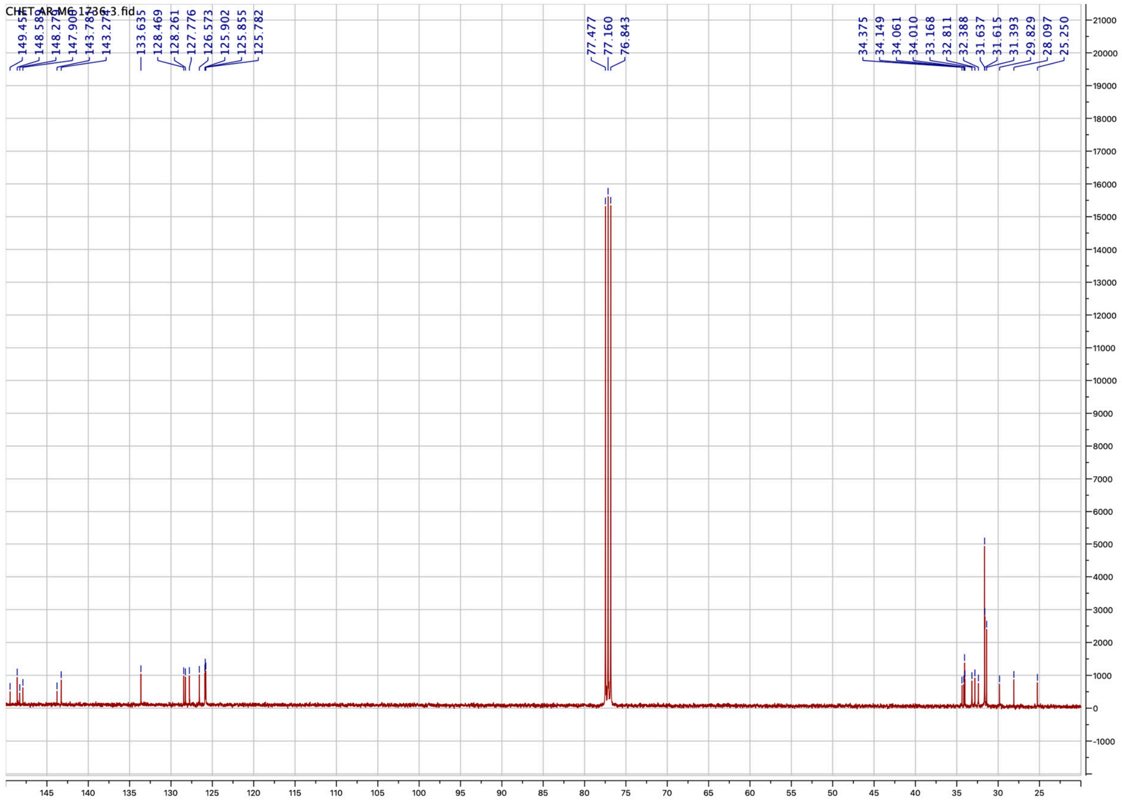

^{1}H NMR spectrum of 4c

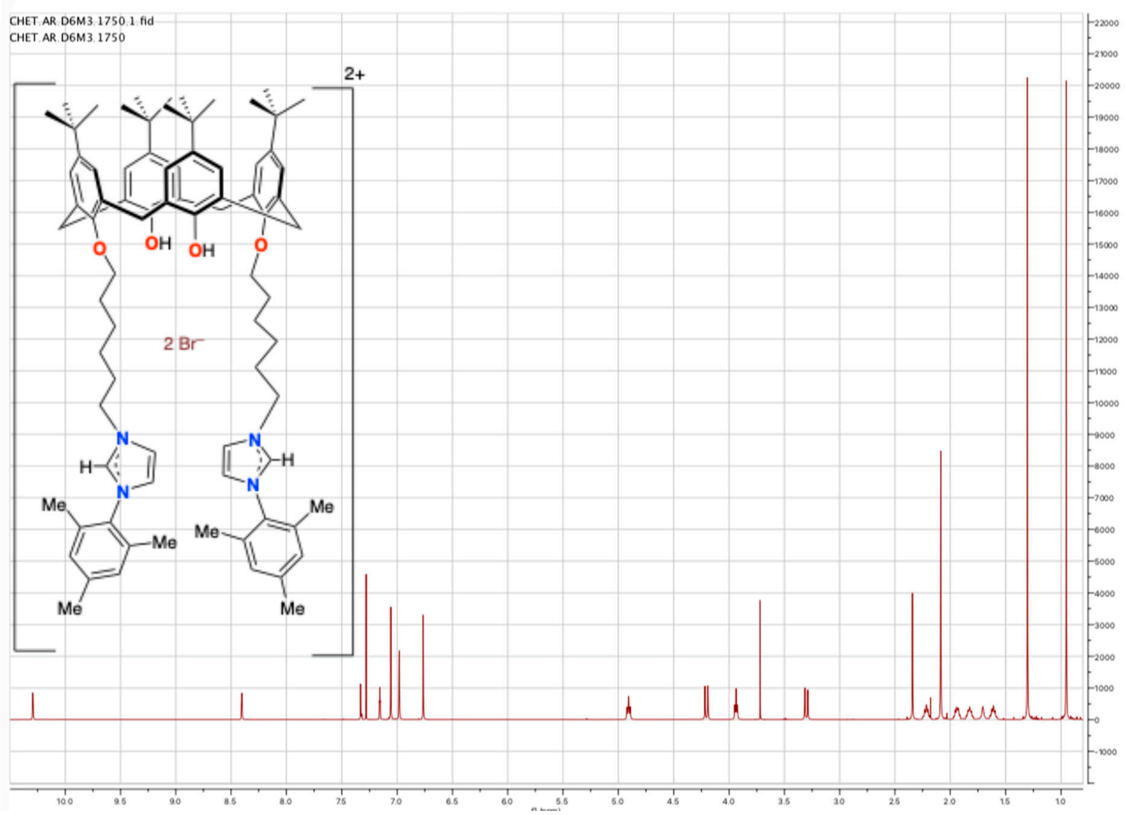

^{13}C NMR spectrum of 4c

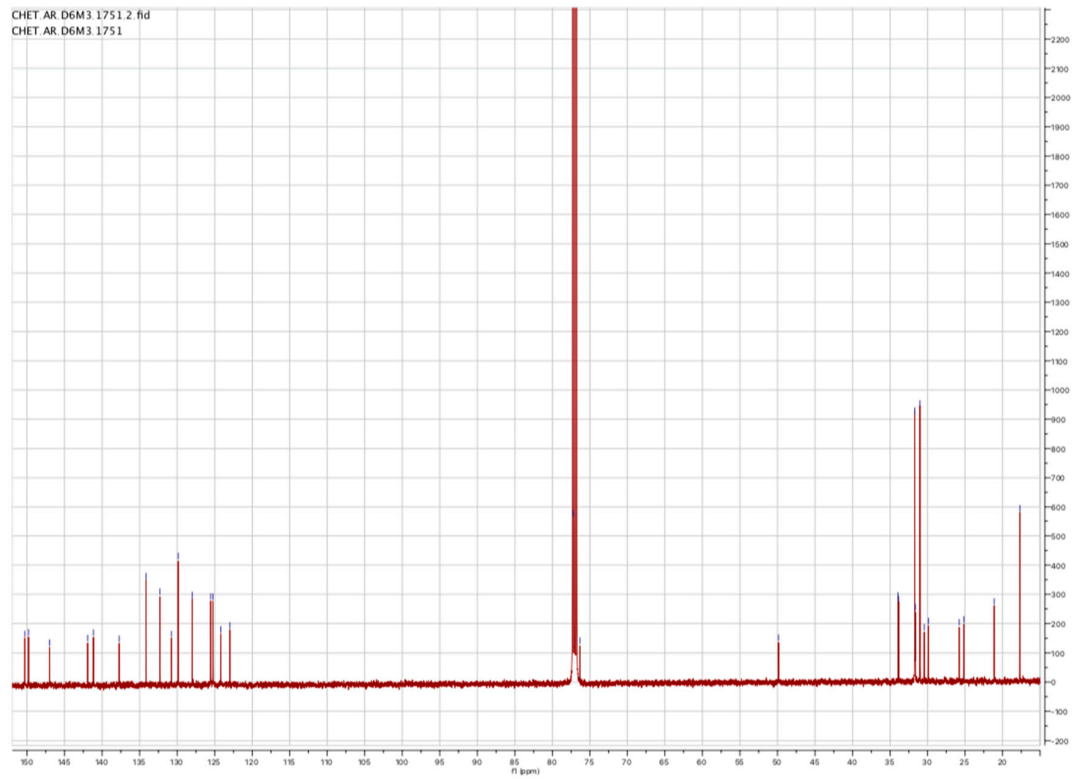

^{1}H NMR spectrum of 4d

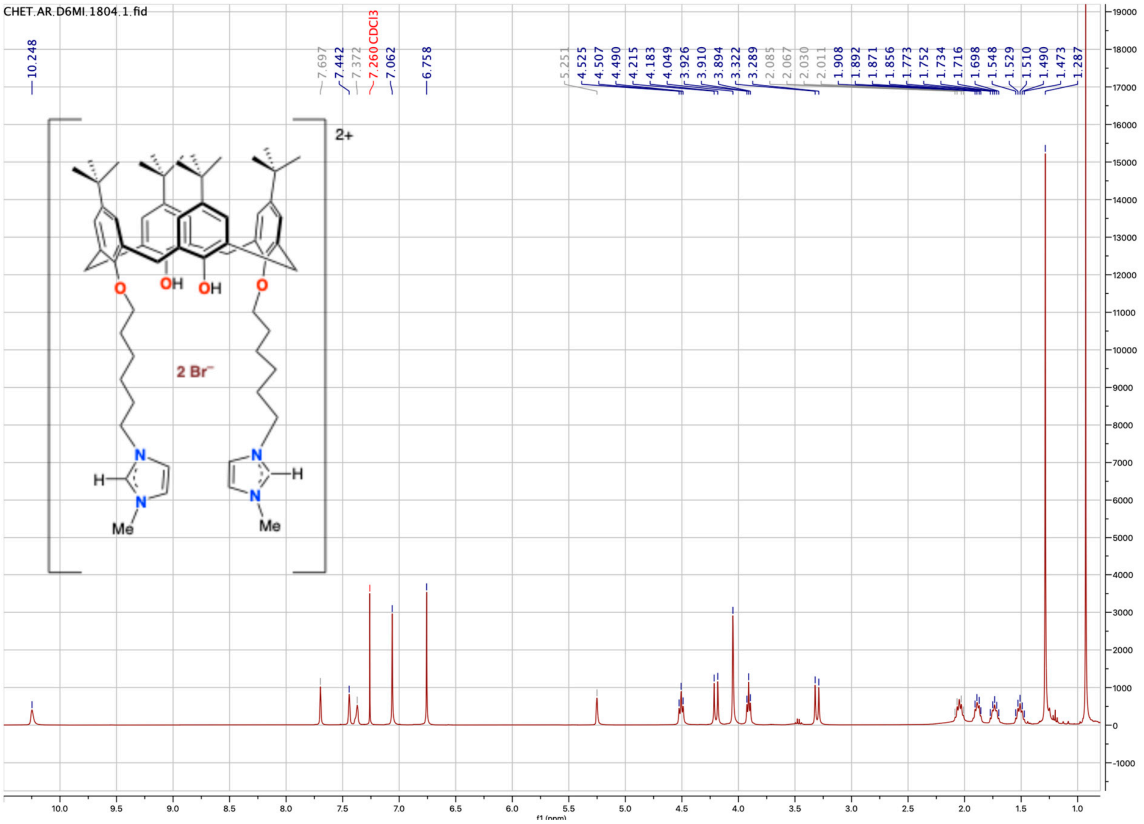

^{13}C NMR spectrum of 4d.

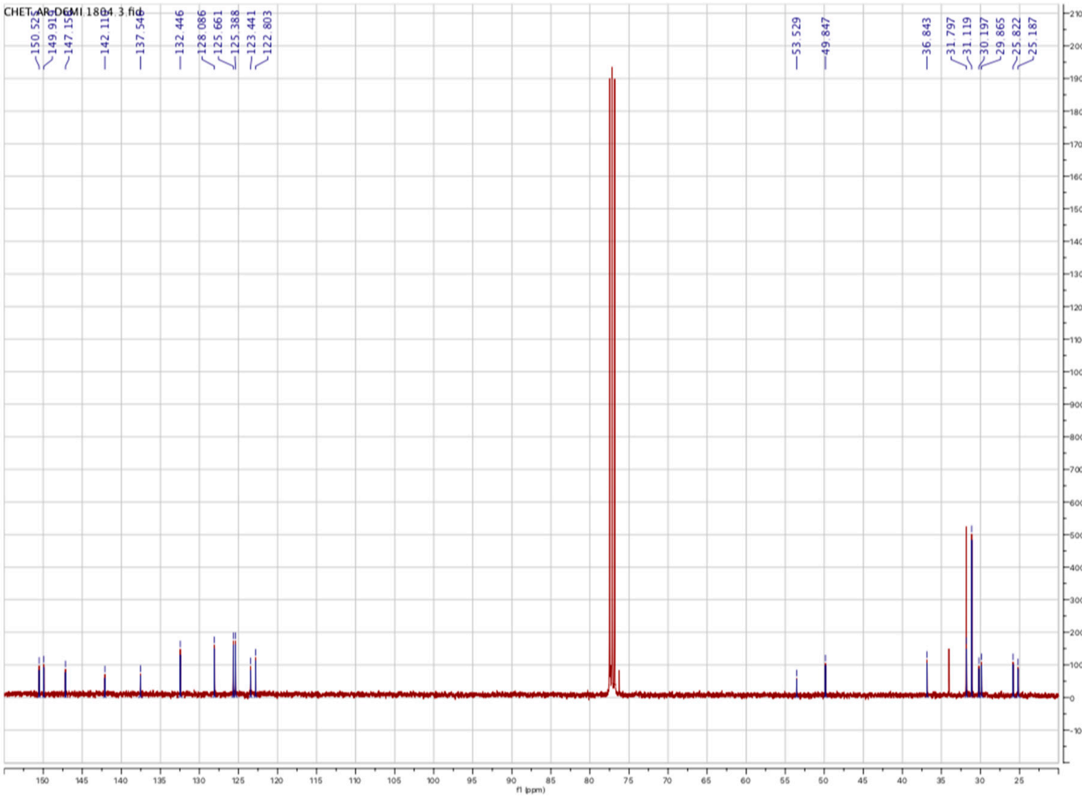

^{1}H NMR spectrum of 5a

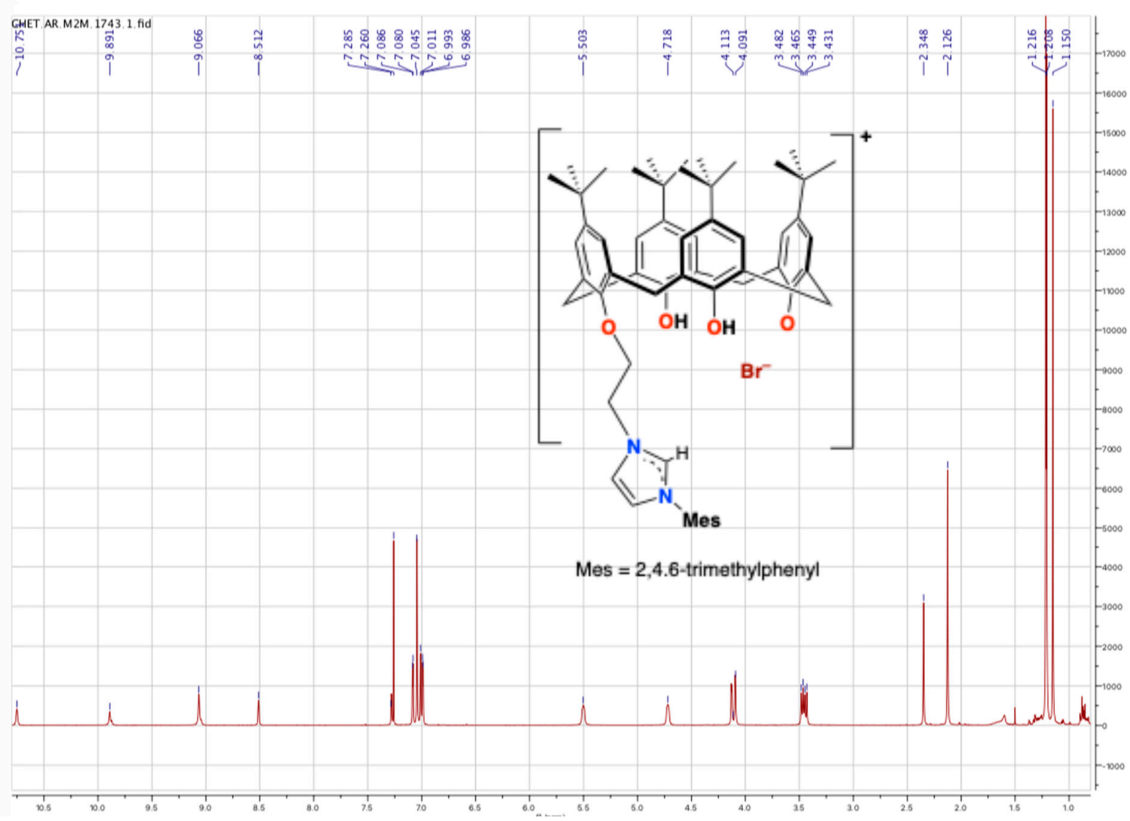

^{13}C NMR spectrum of 5a

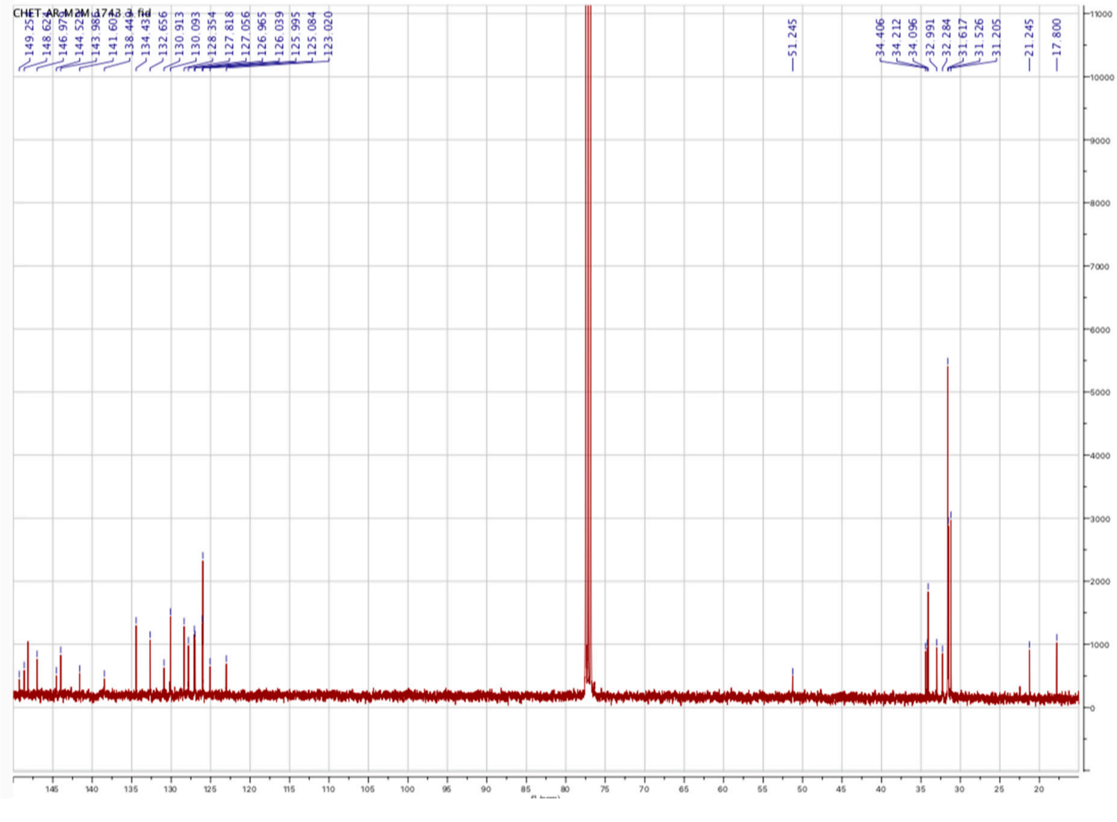

^{1}H NMR spectrum of 5b

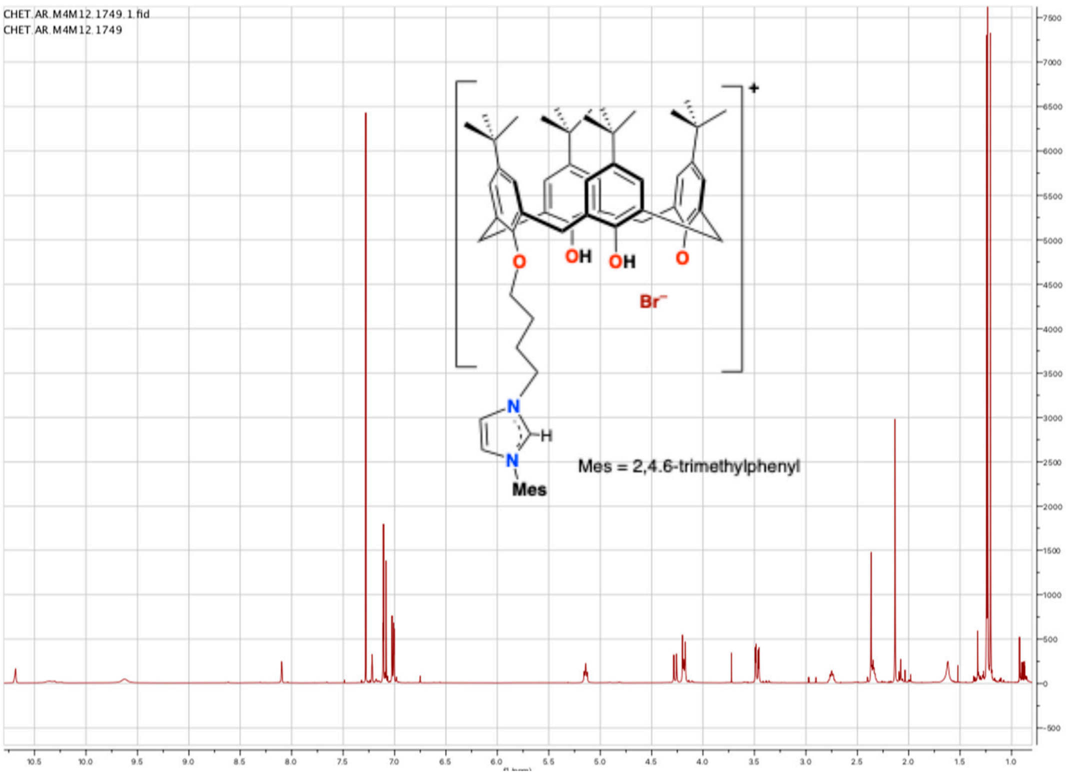

^{13}C NMR spectrum of 5b

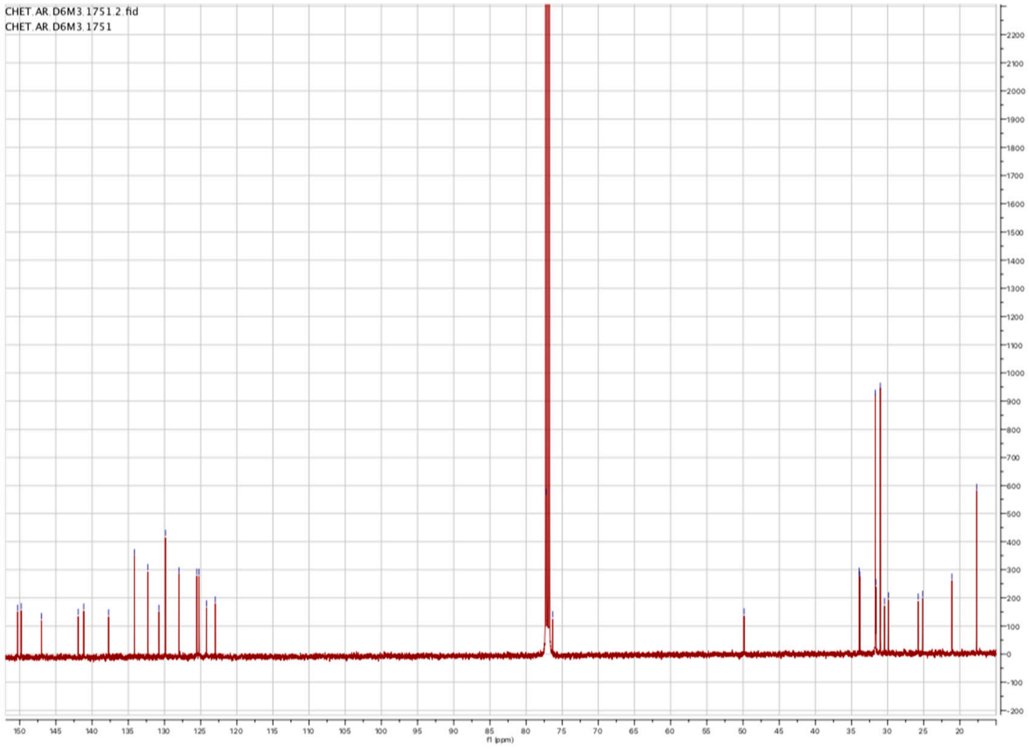

^{1}H NMR spectrum of 5c

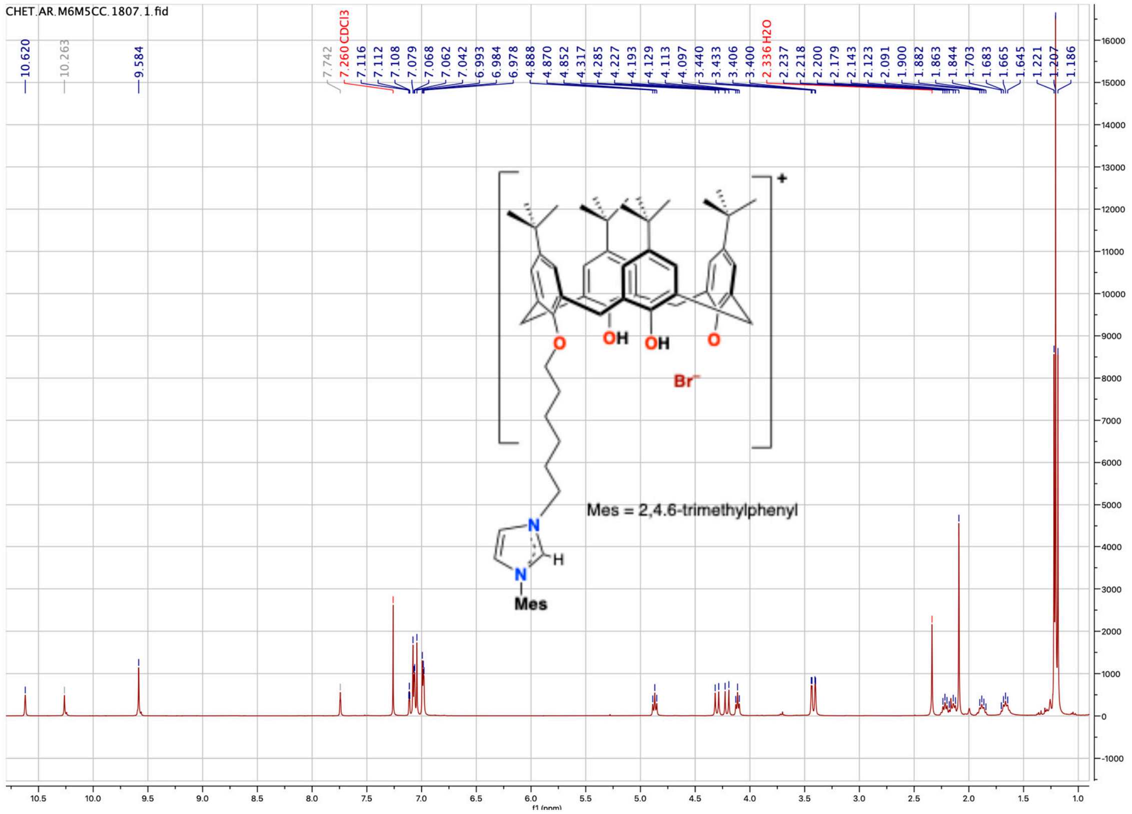

^{13}C NMR spectrum of 5c

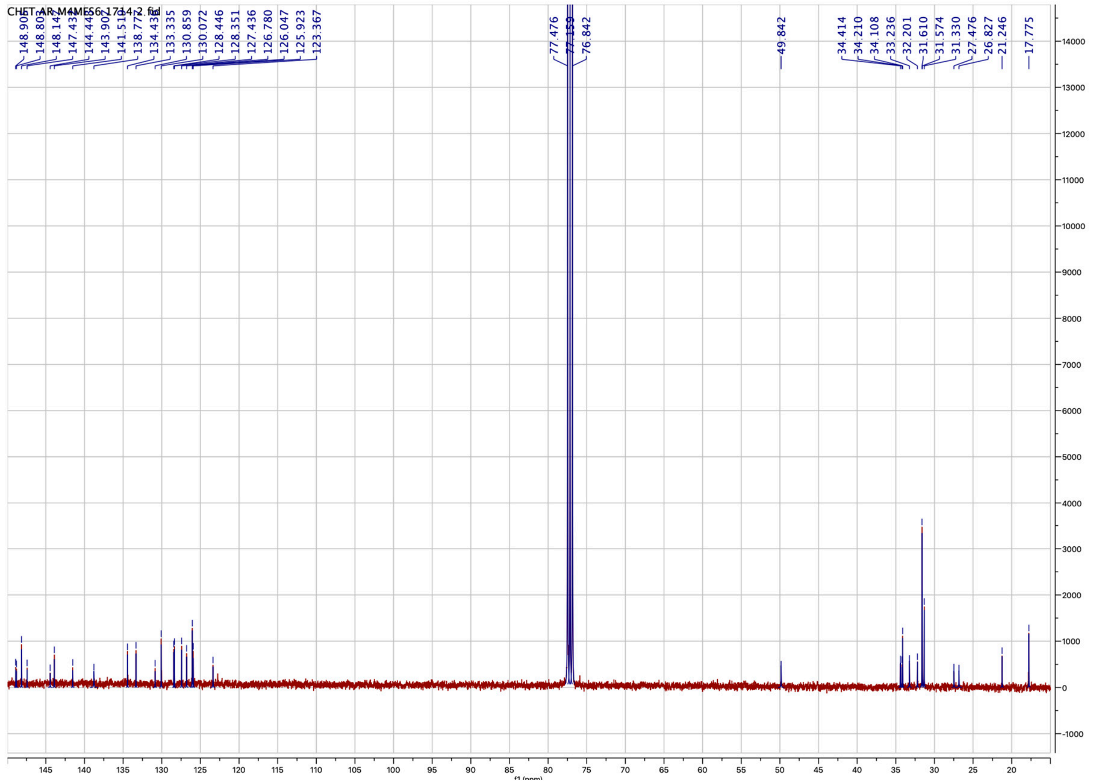

^1H NMR spectrum of 5d

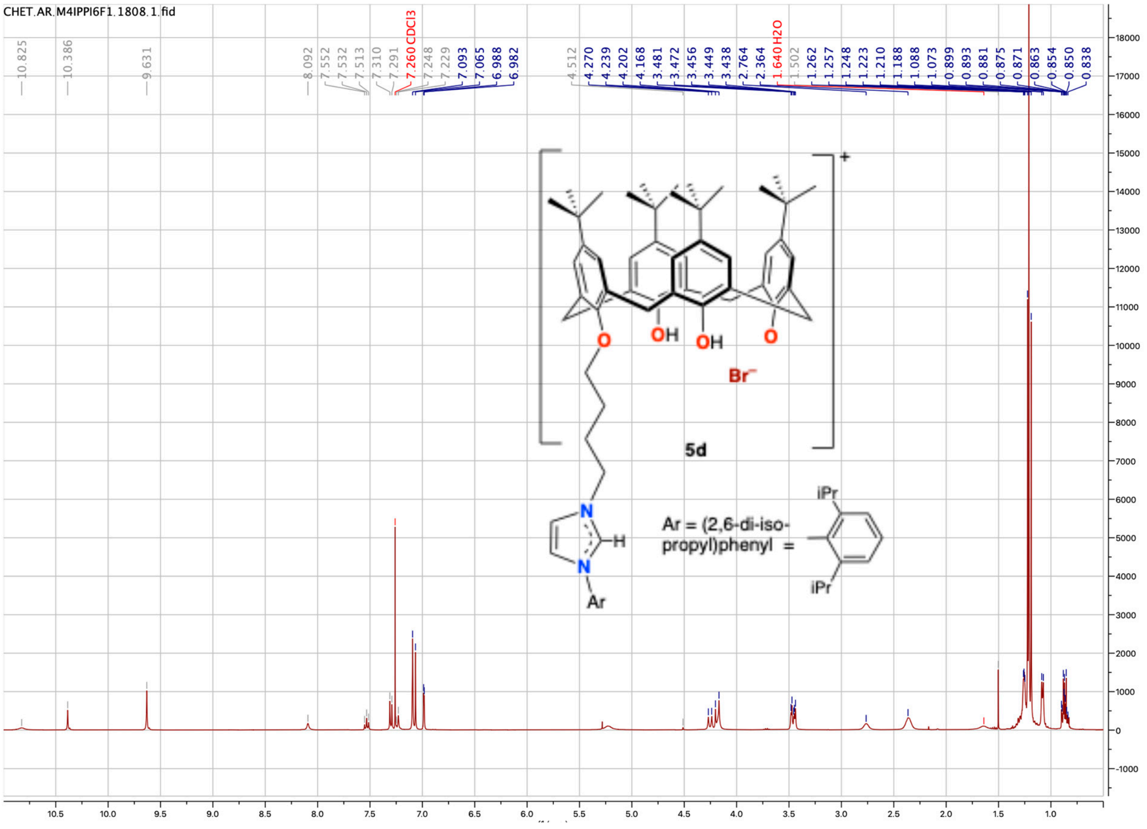

^{13}C NMR spectrum of 5d

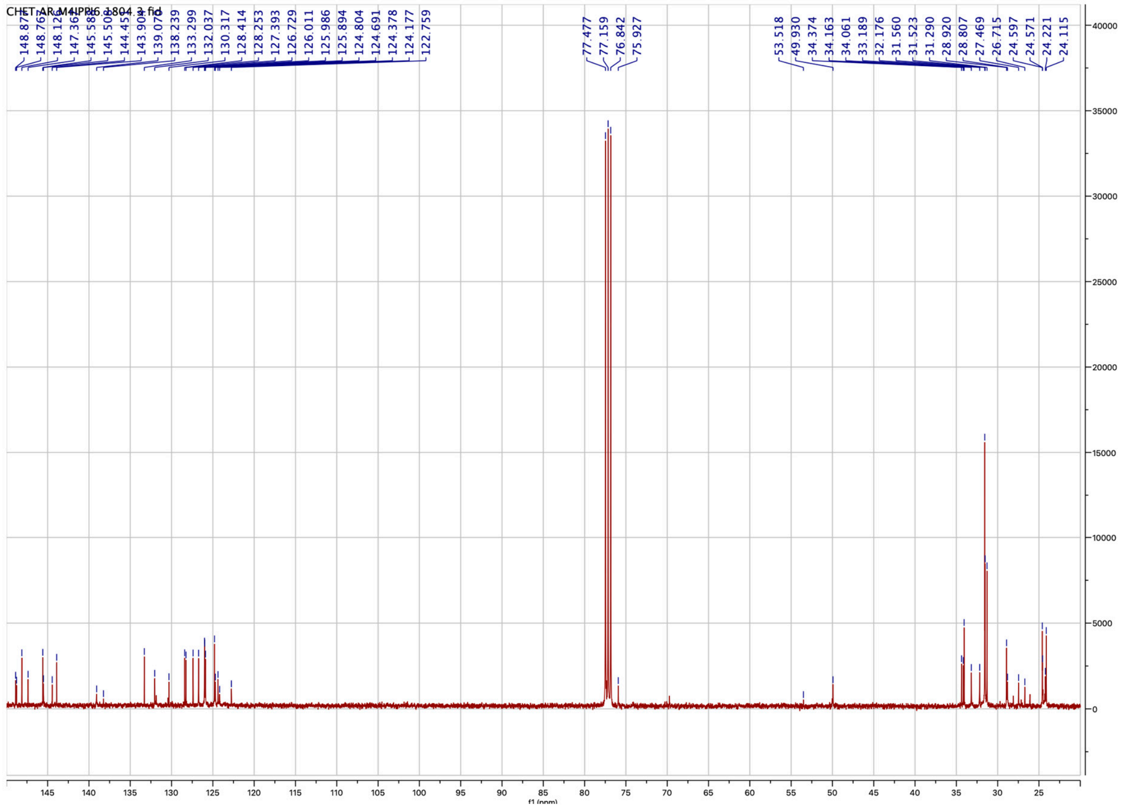

^1H NMR spectrum of **7**

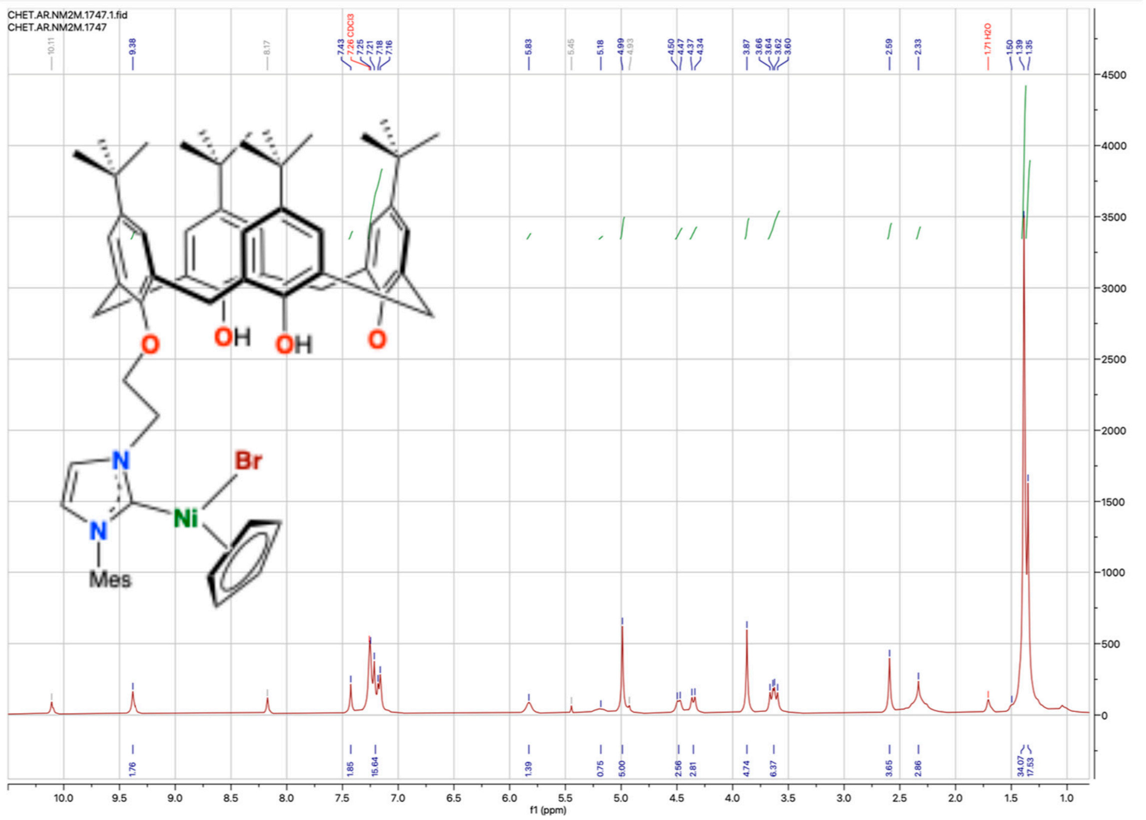

^{13}C NMR spectrum of **7**

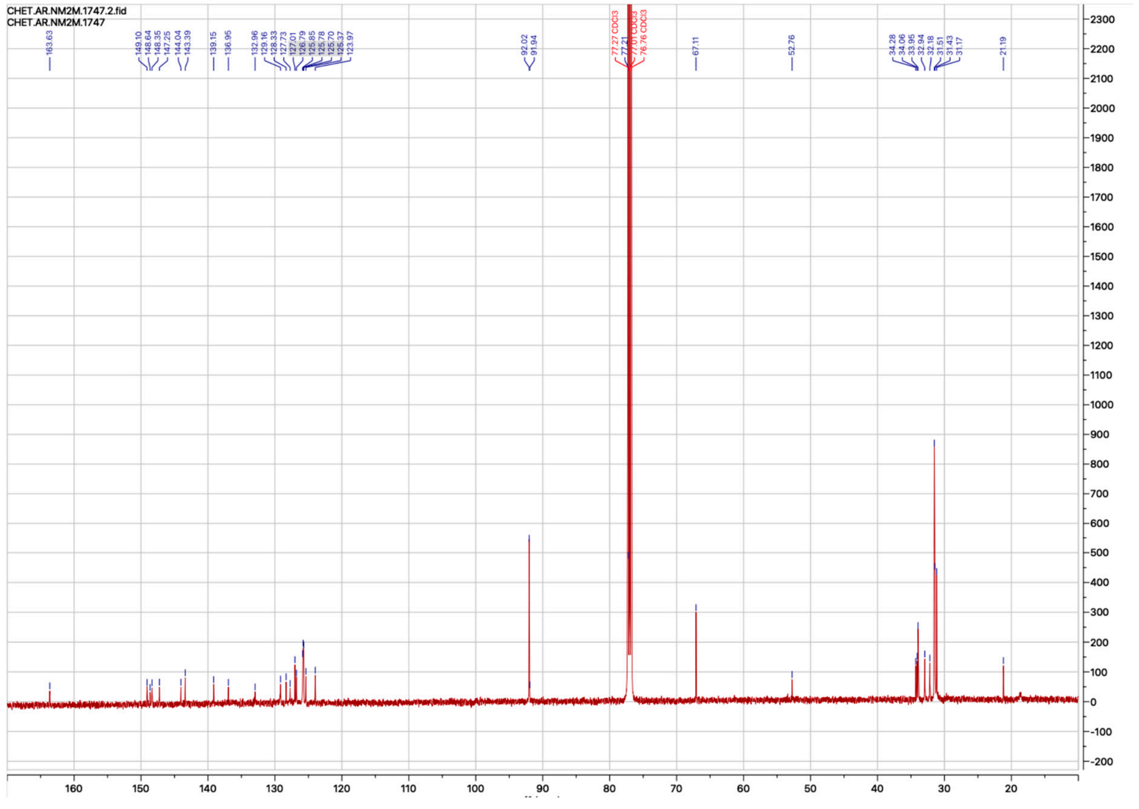

CheckCif for 2

checkCIF (basic structural check) running

Checking for embedded fcf data in CIF ...
Found embedded fcf data in CIF. Extracting fcf data from uploaded CIF, please wait . . . . . .

checkCIF/PLATON (basic structural check)

Structure factors have been supplied for datablock(s) mcra160927

THIS REPORT IS FOR GUIDANCE ONLY. IF USED AS PART OF A REVIEW PROCEDURE FOR PUBLICATION, IT SHOULD NOT REPLACE THE EXPERTISE OF AN EXPERIENCED CRYSTALLOGRAPHIC REFEREE.

No syntax errors found. CIF dictionary
Please wait while processing .... Interpreting this report

Structure factor report

Datablock: mcra160927

```
Bond precision:        C-C = 0.0092 A              Wavelength=1.54178
Cell:        a=12.7460(6)      b=19.0539(8)     c=20.7181(9)
             alpha=84.468(3)   beta=89.930(3)   gamma=80.391(3)
Temperature: 173 K
                    Calculated                       Reported
Volume              4937.4(4)                        4937.4(4)
Space group         P -1                             P -1
Hall group          -P 1                             -P 1
Moiety formula      C92 H116 O8, 4(C H2 Cl2)         C92 H116 O8, 4(C H2 Cl2)
Sum formula         C96 H124 Cl8 O8                  C96 H124 Cl8 O8
Mr                  1689.56                          1689.54
Dx,g cm-3           1.137                            1.136
Z                   2                                2
Mu (mm-1)           2.474                            2.474
F000                1800.0                           1800.0
F000'               1809.97
h,k,lmax            15,22,24                         15,22,24
Nref                17550                            17237
Tmin,Tmax           0.351,0.610                      0.506,0.753
Tmin'               0.252
Correction method= # Reported T Limits: Tmin=0.506 Tmax=0.753
AbsCorr = MULTI-SCAN
Data completeness= 0.982           Theta(max)= 66.854
R(reflections)= 0.1387( 11905)               wR2(reflections)= 0.3672(
                                             17237)
S = 1.023                  Npar= 1048
```

The following ALERTS were generated. Each ALERT has the format
test-name_ALERT_alert-type_alert-level.
Click on the hyperlinks for more details of the test.

Alert level A

PLAT601_ALERT_2_A Unit Cell Contains Solvent Accessible VOIDS <= 259 Ang**3

Alert level B

PLAT084_ALERT_3_B High wR2 Value (i.e. > 0.25) .... 0.37 Report

Alert level C

DIFMX02_ALERT_1_C The maximum difference density is > 0.1*ZMAX*0.75
The relevant atom site should be identified.
PLAT018_ALERT_1_C _diffrn_measured_fraction_theta_max .NE. *_full ! Check
PLAT082_ALERT_2_C High R1 Value .... 0.14 Report
PLAT097_ALERT_2_C Large Reported Max. (Positive) Residual Density 1.42 eA-3
PLAT220_ALERT_2_C NonSolvent Resd 1 C Ueq(max)/Ueq(min) Range 3.4 Ratio
PLAT222_ALERT_3_C NonSolvent Resd 1 H Uiso(max)/Uiso(min) Range 4.3 Ratio
PLAT234_ALERT_4_C Large Hirshfeld Difference C33 --C36B . 0.19 Ang.
And 12 other PLAT234 Alerts
More ...
PLAT242_ALERT_2_C Low 'MainMol' Ueq as Compared to Neighbors of C29 Check
And 7 other PLAT242 Alerts
More ...
PLAT244_ALERT_4_C Low 'Solvent' Ueq as Compared to Neighbors of C93 Check
PLAT244_ALERT_4_C Low 'Solvent' Ueq as Compared to Neighbors of C96 Check
PLAT260_ALERT_2_C Large Average Ueq of Residue Including Cl1 0.118 Check
PLAT260_ALERT_2_C Large Average Ueq of Residue Including Cl7 0.127 Check
PLAT336_ALERT_2_C Long Bond Distance for ..... C96 -Cl7 1.876 Ang.
PLAT336_ALERT_2_C Long Bond Distance for ..... C96 -Cl8B 1.900 Ang.
PLAT340_ALERT_3_C Low Bond Precision on C-C Bonds .... 0.00921 Ang.
PLAT414_ALERT_2_C Short Intra D-H..H-X H2A ..H14B . 1.97 Ang.
x,y,z = 1_555 Check
PLAT906_ALERT_3_C Large K Value in the Analysis of Variance ...... 14.236 Check
PLAT906_ALERT_3_C Large K Value in the Analysis of Variance ...... 3.724 Check
PLAT911_ALERT_3_C Missing FCF Refl Between Thmin & STh/L= 0.596 311 Report
7 2 0, 7 4 0, -4 6 0, 13 11 0, 13 12 0, -1 22 0,
-5-22 1, 3-21 1, -11-18 1, -13-13 1, -12-13 1, -14-12 1,
-15 -7 1, 4 -4 1, 15 7 1, -8 17 1, 8 21 1, -5-22 2,
-4-22 2, -10-19 2, 13 -8 2, -2 2 2, 15 7 2, -4-22 3,
-3-22 3, -7-21 3, 5 -4 3, 0 2 3, -14 5 3, 15 6 3,
(281 More Missing: see the .ckf listing file)

Alert level G

PLAT002_ALERT_2_G Number of Distance or Angle Restraints on AtSite 3 Note
PLAT007_ALERT_5_G Number of Unrefined Donor-H Atoms .... 4 Report
H2A H4A H6 H8A
PLAT083_ALERT_2_G SHELXL Second Parameter in WGHT Unusually Large 28.13 Why ?
PLAT154_ALERT_1_G The s.u.'s on the Cell Angles are Equal ..(Note) 0.003 Degree
PLAT171_ALERT_4_G The CIF-Embedded .res File Contains EADP Records 14 Report
PLAT172_ALERT_4_G The CIF-Embedded .res File Contains DFIX Records 1 Report
PLAT230_ALERT_2_G Hirshfeld Test Diff for C29 --C30 . 8.0 s.u.
And 34 other PLAT230 Alerts
More ...
PLAT299_ALERT_4_G Atom Site Occupancy Constrained at .... 0.5 Check
C42 C42B C43 C43B C44 C44B C91 C91B
C92 C92B H42A H42B H42C H42D H42E H42F
H43A H43B H43C H43D H43E H43F H44A H44B
H44C H44D H44E H44F H91A H91B H91C H91D
H92A H92B H92C H92D Cl1 Cl1B Cl2 Cl2B
H93A H93B H93C H93D Cl7 Cl7B Cl8 Cl8B
PLAT300_ALERT_4_G Atom Site Occupancy of C30 Constrained at 0.6 Check
And 179 other PLAT300 Alerts
More ...
PLAT301_ALERT_3_G Main Residue Disorder ....(Resd 1) 28% Note
PLAT302_ALERT_4_G Anion/Solvent/Minor-Residue Disorder (Resd 2) 67% Note

PLAT302_ALERT_4_G Anion/Solvent/Minor-Residue Disorder (Resd 3) 67% Note
PLAT410_ALERT_2_G Short Intra H...H Contact H21A ..H91B . 2.13 Ang.
x,y,z = 1_555 Check

And 3 other PLAT410 Alerts

More ...

PLAT412_ALERT_2_G Short Intra XH3 .. XHn H2 ..H32B . 1.88 Ang.
x,y,z = 1_555 Check

And 16 other PLAT412 Alerts

More ...

PLAT773_ALERT_2_G Check long C-C Bond in CIF: C75 --C77B 1.71 Ang.
PLAT811_ALERT_5_G No ADDSYM Analysis: Too Many Excluded Atoms .... ! Info
PLAT860_ALERT_3_G Number of Least-Squares Restraints .... 2 Note
PLAT909_ALERT_3_G Percentage of I>2sig(I) Data at Theta(Max) Still 36% Note
PLAT910_ALERT_3_G Missing FCF Reflection(s) Below Theta(Min)[Deg]= 3.03 Note
0 1 0, 0 0 1,
PLAT913_ALERT_3_G Missing # of Very Strong Reflections in FCF .... 1 Note
0 1 0,
PLAT941_ALERT_3_G Average HKL Measurement Multiplicity .... 4.3 Low
PLAT969_ALERT_5_G The 'Henn et al.' R-Factor-gap value .... 4.740 Note
Predicted wR2: Based on SigI**2 7.75 or SHELX Weight 35.89
PLAT978_ALERT_2_G Number C-C Bonds with Positive Residual Density. 0 Info

---

1 **ALERT level A** = Most likely a serious problem - resolve or explain
1 **ALERT level B** = A potentially serious problem, consider carefully
38 **ALERT level C** = Check. Ensure it is not caused by an omission or oversight
255 **ALERT level G** = General information/check it is not something unexpected

3 ALERT type 1 CIF construction/syntax error, inconsistent or missing data
77 ALERT type 2 Indicator that the structure model may be wrong or deficient
12 ALERT type 3 Indicator that the structure quality may be low
200 ALERT type 4 Improvement, methodology, query or suggestion
3 ALERT type 5 Informative message, check

It is advisable to attempt to resolve as many as possible of the alerts in all categories. Often the minor alerts point to easily fixed oversights, errors and omissions in your CIF or refinement strategy, so attention to these fine details can be worthwhile. In order to resolve some of the more serious problems it may be necessary to carry out additional measurements or structure refinements. However, the purpose of your study may justify the reported deviations and the more serious of these should normally be commented upon in the discussion or experimental section of a paper or in the "special_details" fields of the CIF. checkCIF was carefully designed to identify outliers and unusual parameters, but every test has its limitations and alerts that are not important in a particular case may appear. Conversely, the absence of alerts does not guarantee there are no aspects of the results needing attention. It is up to the individual to critically assess their own results and, if necessary, seek expert advice.

Publication of your CIF in IUCr journals

A basic structural check has been run on your CIF. These basic checks will be run on all CIFs submitted for publication in IUCr journals (*Acta Crystallographica*, *Journal of Applied Crystallography*, *Journal of Synchrotron Radiation*); however, if you intend to submit to *Acta Crystallographica Section C* or *E* or *IUCrData*, you should make sure that full publication checks are run on the final version of your CIF prior to submission.

Publication of your CIF in other journals

Please refer to the *Notes for Authors* of the relevant journal for any special instructions relating to CIF submission.

PLATON version of 04/06/2025; check.def file version of 30/05/2025

Datablock mcra160927 - ellipsoid plot

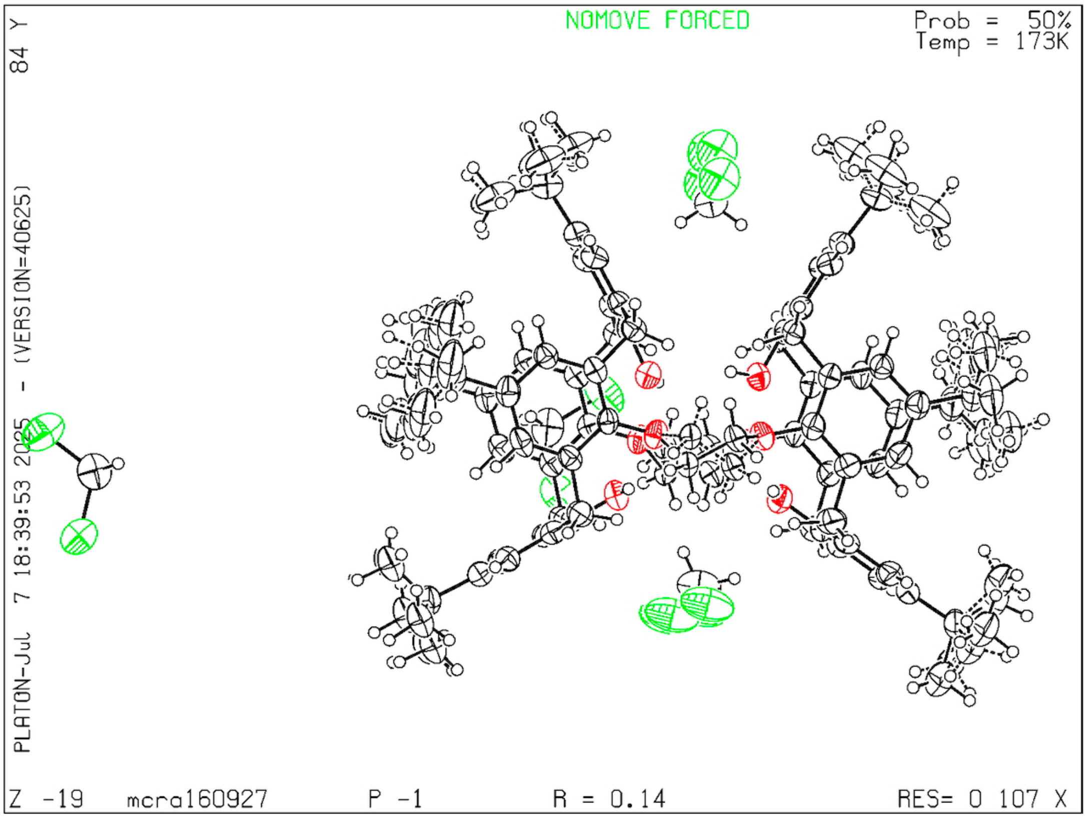

Most alerts in the CheckCif are the result of small crystals, poor crystal quality and very significant disorder.

CheckCif for 6

checkCIF (basic structural check) running

Checking for embedded fcf data in CIF ...
Found embedded fcf data in CIF. Extracting fcf data from uploaded CIF, please wait . . . . . .

checkCIF/PLATON (basic structural check)

Structure factors have been supplied for datablock(s) mcra160405

THIS REPORT IS FOR GUIDANCE ONLY. IF USED AS PART OF A REVIEW PROCEDURE FOR PUBLICATION, IT SHOULD NOT REPLACE THE EXPERTISE OF AN EXPERIENCED CRYSTALLOGRAPHIC REFEREE.

No syntax errors found. CIF dictionary
Please wait while processing .... Interpreting this report
Structure factor report

Datablock: mcra160405

```
Bond precision:     C-C = 0.0096 A            Wavelength=1.54178
Cell:         a=12.9974(8)    b=20.4956(15)   c=38.955(3)
              alpha=90        beta=96.632(4)  gamma=90
Temperature:173 K
```

	Calculated	Reported
Volume	10307.8(13)	10307.7(12)
Space group	P 21/c	P 21/c
Hall group	-P 2ybc	-P 2ybc
Moiety formula	C99 H129 N2 O8, 2(C H4 O) [+ solvent]	C99 H129 N2 O8, 2(C H4 O)
Sum formula	C101 H137 N2 O10 [+ solvent]	C101 H137 N2 O10
Mr	1539.13	1539.12
Dx,g cm-3	0.992	0.992
Z	4	4
Mu (mm-1)	0.487	0.487
F000	3348.0	3348.0
F000'	3357.11	
h,k,lmax	15,24,46	15,24,46
Nref	18369	17146
Tmin,Tmax	0.916,0.952	0.609,0.753
Tmin'	0.864	

```
Correction method= # Reported T Limits: Tmin=0.609
Tmax=0.753 AbsCorr = MULTI-SCAN
Data completeness= 0.933        Theta(max)= 66.955
```

R(reflections)= 0.1388(7558)   wR2(reflections)= 0.3812(17146)

S = 1.067   Npar= 1030

The following ALERTS were generated. Each ALERT has the format
test-name_ALERT_alert-type_alert-level.
Click on the hyperlinks for more details of the test.

Alert level B

PLAT084_ALERT_3_B High wR2 Value (i.e. > 0.25) .... 0.38 Report
PLAT230_ALERT_2_B Hirshfeld Test Diff for C37 --C38 . 20.5 s.u.
PLAT230_ALERT_2_B Hirshfeld Test Diff for C37 --C39 . 11.2 s.u.
PLAT242_ALERT_2_B Low 'MainMol' Ueq as Compared to Neighbors of C37 Check
PLAT412_ALERT_2_B Short Intra XH3 .. XHn H38C ..H40B . 1.74 Ang.
x,y,z = 1_555 Check
PLAT911_ALERT_3_B Missing FCF Refl Between Thmin & STh/L= 0.597 1223 Report
15 0 0, 15 1 0, 15 2 0, 15 3 0, 15 4 0, 14 10 0,
13 13 0, 12 15 0, 2 16 0, 1 17 0, 2 17 0, 11 17 0,
0 18 0, 1 18 0, 2 18 0, 1 19 0, 2 19 0, 3 19 0,
0 20 0, 1 20 0, 2 20 0, 3 20 0, 1 21 0, 2 21 0,
3 21 0, 0 22 0, 1 22 0, 2 22 0, -15 1 1, 15 1 1,
(1193 More Missing: see the .ckf listing file)
PLAT990_ALERT_1_B Deprecated .res/.hkl Input Style SQUEEZE Job ... ! Note

Alert level C

PLAT018_ALERT_1_C _diffrn_measured_fraction_theta_max .NE. *_full ! Check
PLAT026_ALERT_3_C Ratio Observed / Unique Reflections (too) Low .. 44% Check
PLAT082_ALERT_2_C High R1 Value .... 0.14 Report
PLAT220_ALERT_2_C NonSolvent Resd 1 C Ueq(max)/Ueq(min) Range 4.0 Ratio
PLAT222_ALERT_3_C NonSolvent Resd 1 H Uiso(max)/Uiso(min) Range 5.9 Ratio
PLAT230_ALERT_2_C Hirshfeld Test Diff for C33 --C35 . 5.3 s.u.
PLAT230_ALERT_2_C Hirshfeld Test Diff for C79 --C80 . 5.5 s.u.
PLAT234_ALERT_4_C Large Hirshfeld Difference C4 --C29 . 0.18 Ang.
And 16 other PLAT234 Alerts
More ...
PLAT242_ALERT_2_C Low 'MainMol' Ueq as Compared to Neighbors of C29 Check
And 6 other PLAT242 Alerts
More ...
PLAT245_ALERT_2_C U(iso) H6 Smaller than U(eq) O6 by 0.022 Ang**2
And 3 other PLAT245 Alerts
More ...
PLAT260_ALERT_2_C Large Average Ueq of Residue Including O9 0.106 Check
PLAT340_ALERT_3_C Low Bond Precision on C-C Bonds .... 0.00959 Ang.
PLAT414_ALERT_2_C Short Intra D-H..H-X H3A ..H14A . 1.92 Ang.
x,y,z = 1_555 Check
PLAT414_ALERT_2_C Short Intra D-H..H-X H8 ..H83B . 1.95 Ang.
x,y,z = 1_555 Check
PLAT906_ALERT_3_C Large K Value in the Analysis of Variance ...... 35.450 Check
And 2 other PLAT906 Alerts
More ...
PLAT977_ALERT_2_C Check Negative Difference Density on H90D . -0.31 eA-3

Alert level G

PLAT002_ALERT_2_G Number of Distance or Angle Restraints on AtSite 26 Note
PLAT007_ALERT_5_G Number of Unrefined Donor-H Atoms .... 8 Report
H2 H3A H4 H6 H7C H8 H9A H10G

PLAT169_ALERT_4_G The CIF-Embedded .res File Contains AFIX 1 Recds 1 Report
PLAT171_ALERT_4_G The CIF-Embedded .res File Contains EADP Records 9 Report
PLAT172_ALERT_4_G The CIF-Embedded .res File Contains DFIX Records 22 Report
PLAT230_ALERT_2_G Hirshfeld Test Diff for C29 --C32 . 5.8 s.u.
And 13 other PLAT230 Alerts
More ...
PLAT299_ALERT_4_G Atom Site Occupancy Constrained at .... 0.5 Check
C42 C42B C43 C43B C44 C44B H42A H42B
H42C H42D H42E H42F H43A H43B H43C H43D
H43E H43F H44A H44B H44C H44D H44E H44F
PLAT300_ALERT_4_G Atom Site Occupancy of C30 Constrained at 0.65 Check
And 119 other PLAT300 Alerts
More ...
PLAT301_ALERT_3_G Main Residue Disorder ....(Resd 1) 17% Note
PLAT398_ALERT_2_G Deviating C-O-C Angle From 120 for O1 . 109.9 Degree
PLAT412_ALERT_2_G Short Intra XH3 .. XHn H5 ..H31D . 1.95 Ang.
x,y,z = 1_555 Check
And 14 other PLAT412 Alerts
More ...
PLAT413_ALERT_2_G Short Inter XH3 .. XHn H39B ..H98F . 1.92 Ang.
x,1/2-y,1/2+z = 4_566 Check
PLAT606_ALERT_4_G Solvent Accessible VOID(S) in Crystal Structure ! Info
PLAT802_ALERT_4_G CIF Input Record(s) with more than 80 Characters 1 Info
PLAT860_ALERT_3_G Number of Least-Squares Restraints .... 22 Note
PLAT869_ALERT_4_G ALERTS Related to the Use of SQUEEZE Suppressed ! Info
PLAT941_ALERT_3_G Average HKL Measurement Multiplicity .... 2.8 Low
PLAT961_ALERT_5_G Dataset Contains no Negative Intensities .... Please Check
PLAT969_ALERT_5_G The 'Henn et al.' R-Factor-gap value .... 3.566 Note
Predicted wR2: Based on SigI**2 10.69 or SHELX Weight 35.74
PLAT978_ALERT_2_G Number C-C Bonds with Positive Residual Density. 0 Info

---

0 **ALERT level A** = Most likely a serious problem - resolve or explain
7 **ALERT level B** = A potentially serious problem, consider carefully
43 **ALERT level C** = Check. Ensure it is not caused by an omission or oversight
166 **ALERT level G** = General information/check it is not something unexpected

2 ALERT type 1 CIF construction/syntax error, inconsistent or missing data
56 ALERT type 2 Indicator that the structure model may be wrong or deficient
11 ALERT type 3 Indicator that the structure quality may be low
144 ALERT type 4 Improvement, methodology, query or suggestion
3 ALERT type 5 Informative message, check

It is advisable to attempt to resolve as many as possible of the alerts in all categories. Often the minor alerts point to easily fixed oversights, errors and omissions in your CIF or refinement strategy, so attention to these fine details can be worthwhile. In order to resolve some of the more serious problems it may be necessary to carry out additional measurements or structure refinements. However, the purpose of your study may justify the reported deviations and the more serious of these should normally be commented upon in the discussion or experimental section of a paper or in the "special_details" fields of the CIF. checkCIF was carefully designed to identify outliers and unusual parameters, but every test has its limitations and alerts that are not important in a particular case may appear. Conversely, the absence of alerts does not guarantee there are no aspects of the results needing attention. It is up to the individual to critically assess their own results and, if necessary, seek expert advice.

Publication of your CIF in IUCr journals

A basic structural check has been run on your CIF. These basic checks will be run on all CIFs submitted for publication in IUCr journals (*Acta Crystallographica*, *Journal of Applied Crystallography*, *Journal of Synchrotron Radiation*); however, if you intend to submit to *Acta Crystallographica Section C* or *E* or *IUCrData*, you should make sure that full publication checks are run on the final version of your CIF prior to submission.

Publication of your CIF in other journals

Please refer to the *Notes for Authors* of the relevant journal for any special instructions relating to CIF submission.

PLATON version of 04/06/2025; check.def file version of 30/05/2025

Datablock mcra160405 - ellipsoid plot

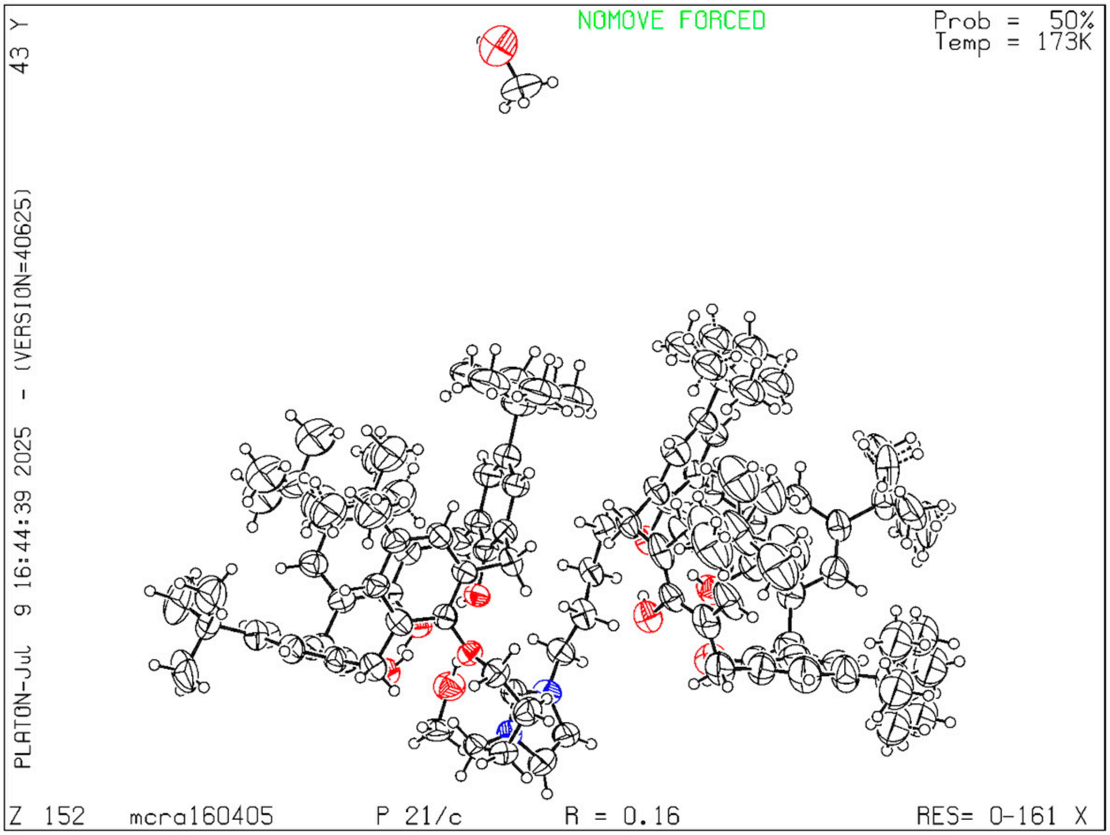

Download CIF editor (publCIF) from the IUCr
Download CIF editor (enCIFer) from the CCDC
Test a new CIF entry

The many alerts in the CheckCif are the result of small crystals, poor crystal quality and very significant disorder.
